# Treatment in certified cancer centers is related to better survival in patients with colon and rectal cancer: evidence from a large German cohort study

**DOI:** 10.1186/s12957-023-03262-9

**Published:** 2024-01-06

**Authors:** Veronika Bierbaum, Christoph Bobeth, Martin Roessler, Michael Gerken, Kees Kleihues-van Tol, Christoph Reissfelder, Alois Fürst, Christian Günster, Patrik Dröge, Thomas Ruhnke, Monika Klinkhammer-Schalke, Jochen Schmitt, Olaf Schoffer

**Affiliations:** 1 Zentrum für Evidenzbasierte Gesundheitsversorgung, Universitätsklinikum und Medizinische Fakultät Carl Gustav Carus, TU Dresden, Dresden, Germany; 2 Arbeitsgemeinschaft Deutscher Tumorzentren e.V., Berlin, Germany; 3 Tumorzentrum Regensburg, Zentrum für Qualitätssicherung und Versorgungsforschung an der Fakultät für Medizin der Universität Regensburg, Regensburg, Germany; 4 Chirurgische Klinik, Universitätsmedizin Mannheim, Medizinische Fakultät Mannheim, Universität Heidelberg, Heidelberg, Germany; 5 Klinik für Allgemein-, Viszeral-, Thoraxchirurgie, Adipositasmedizin, Caritas-Krankenhaus St. Josef Regensburg, Regensburg, Germany; 6 AOK Research Institute, Berlin, Germany

**Keywords:** Certified cancer center, Colon cancer, Rectal cancer, Cohort study, Survival, Cox regression, Quality of cancer care

## Abstract

**Background:**

Certified cancer centers aim to ensure high-quality care by establishing structural and procedural standards according to evidence-based guidelines. Despite the high clinical and health policy relevance, evidence from a nation-wide study for the effectiveness of care for colorectal cancer in certified centers vs. other hospitals in Germany is still missing.

**Methods:**

In a retrospective cohort study covering the years 2009–2017, we analyzed patient data using demographic information, diagnoses, and treatments from a nationwide statutory health insurance enriched with information on certification. We investigated whether patients with incident colon or rectal cancer did benefit from primary therapy in a certified cancer center. We used relative survival analysis taking into account mortality data of the German population and adjustment for patient and hospital characteristics via Cox regression with shared frailty for patients in hospitals with and without certification.

**Results:**

The cohorts for colon and rectal cancer consisted of 109,518 and 51,417 patients, respectively, treated in a total of 1052 hospitals. 37.2% of patients with colon and 42.9% of patients with rectal cancer were treated in a certified center. Patient age, sex, comorbidities, secondary malignoma, and distant metastases were similar across groups (certified/non-certified) for both colon and rectal cancer. Relative survival analysis showed significantly better survival of patients treated in a certified center, with 68.3% (non-certified hospitals 65.8%) 5-year survival for treatment of colon cancer in certified (*p* < 0.001) and 65.0% (58.8%) 5-year survival in case of rectal cancer (*p* < 0.001), respectively. Cox regression with adjustment for relevant covariates yielded a lower hazard of death for patients treated in certified centers for both colon (HR = 0.92, 95% CI = 0.89–0.95) and rectal cancer (HR = 0.92, 95% CI = 0.88–0.95). The results remained robust in a series of sensitivity analyses.

**Conclusions:**

This large cohort study yields new important evidence that patients with colorectal cancer have a better chance of survival if treated in a certified cancer center. Certification thus provides one powerful means to improve the quality of care for colorectal cancer. To decrease the burden of disease, more patients should thus receive cancer care in a certified center.

**Supplementary Information:**

The online version contains supplementary material available at 10.1186/s12957-023-03262-9.

## Background

Colorectal cancer is among the most common causes of cancer both worldwide and in Europe [1]. In Germany, incident diagnoses of colon cancer rank second for women and third for men across all cancer types [2]. Especially at low stages and young age, colorectal cancer is associated with a good survival prognosis [1–3]. The treatment of colorectal cancer involves multiple areas of expertise, involving resection, (neo)-adjuvant chemotherapy, radiotherapy, and/or targeted therapy, depending on the UICC stage [4]. Resection is a central part of curative [5] and even palliative [6] therapy, making the quality of resection an integral part of successful treatment. Health systems worldwide seek to ensure high quality of cancer care through either accreditation (USA) or certification (Europe, Germany) of hospitals ([7], https://www.facs.org/quality-programs/cancer-programs/commission-on-cancer/). Quality of care encompasses measures that improve patient outcomes, particularly survival, such as specialization, evidence-based treatment standards, key performance measures such as minimal volume, and a structured approach to interdisciplinary, cross-sectoral treatment provided by mandatory tumor board discussions, with criteria varying across accreditation/certification programs.

The certification of hospitals stipulates the use of evidence-based clinical practice guidelines in that the quality indicators defined in those (S3-) guidelines are used in certified facilities. In accordance with their mandate, colorectal cancer centers pay attention to high guideline adherence and optimal specimen quality of the removed carcinoma. Due to the defined minimum case numbers for specified surgical procedures, it is generally easier for centers to maintain minimally invasive surgical teams. Evaluations of such programs have been increasingly reported internationally over the past years [8–15]. However, certification programs differ, evidence from Germany is missing, and evidence from other programs may not be generalizable to the German cancer center certification program. Certification programs in Germany are covered by different societies such as the German Cancer Society (GCS; German: Deutsche Krebsgesellschaft, DKG) [16], the DGHO (Deutsche Gesellschaft für Hämatologie und Medizinische Onkologie e. V.), and DGAV (Deutsche Gesellschaft für Allgemein- und Viszeralchirurgie e.V.), with the GCS covering the largest fraction of hospitals and cancer types. The certificates issued by the GCS are linked to a set of professional and quality requirements and are based on S3 guidelines [16]. Since 2016, non-German countries have joined the program through the European ECC Initiative which has its foundation in the Certification System of the German Cancer society. There are, as of December 2022, 1905 German Cancer Centers and 158 Centers in Europe, making it the largest in Europe [17]. The program requires annual reports via entity specific surveys and indicator sheets, and the continuity of certification depends upon these. The program structures the entire process of care ranging from multidisciplinary communication, outreach to the outpatient sector, psychoongological interventions, social care and rehabilitation [17]. The colon cancer program has been in place since 2006 and contains detailed reportings about therapy-related measures along with the indicator sheets currently covering 31 key figures, 10 of which are quality indicators as defined by S3 guidelines related to colon and rectal cancer [18–20]. It is hence reasonable to assume that these measures ultimately improve outcomes. Indeed, regional investigations from Germany suggested that treatment in certified hospitals appears to be associated with better outcomes for colon, rectal, prostate, and pancreatic cancer [11, 12, 14, 21], but large, nationwide investigations are still missing, with the exception of pancreatic cancer [19]. Therefore, it is still controversially debated whether certification improves specific surgical outcomes in colon cancer [20]. For breast cancer, studies addressing the effect of certification do not show clear-cut results [22–24]. Previous studies about certification and colorectal cancer have limitations such as either sample size, a limited time range, data from a specific region, or missing relevant covariates at the patient and the hospital level. In this study, we aimed to strengthen the available evidence about potential survival-related differences in certified compared to non-certified hospitals through a nationwide sample including more than 150,000 patients diagnosed with colorectal cancer. Our hypothesis was that patients who have been treated in certified centers have better long-term overall survival than patients who have been treated in non-certified hospitals.

## Methods

### The WiZen study

The results presented here are part of the WiZen (Wirksamkeit der Versorgung in onkologischen Zentren/ Effectiveness of care in oncological centers) study, which was publicly funded by the German Federal Joint Committee (G-BA, Gemeinsamer Bundesausschuss) as part of the Innovationsfonds program to further develop the German healthcare system based on the standards and principles of evidence-based healthcare (funding number 01VSF17020). WiZen is a controlled cohort study based on data from the largest German statutory health insurance (AOK, Allgemeine Ortskrankenkasse), data from four clinical cancer registries, data about hospital certification status and hospital characteristics based on the mandatory, standardized quality reports of German hospitals. The main objective of the study was to compare certified cancer centers (“certified hospital group”) and non-certified hospitals (“non-certified hospital group”) in Germany regarding the survival of patients with at least one of eight cancer entities (colorectal, pancreatic, lung, prostate, breast, head and neck, brain cancer, and gynecological tumors), and to quantify possible survival differences in treatment between certified cancer centers and non-certified hospitals. The study includes a separate analysis of health insurance and cancer registry data, and a linkage resulting in a subset of data that covers as many confounders as possible. In this paper, we present separate analyses for colon (ICD-10 C18-C19) and rectal (ICD-10 C20) cancer using health insurance data as the main source of information. The study has been registered at ClinicalTrials.gov (ID: NCT04334239) and is reported in agreement with the STROBE requirements and the German Standard for Reporting of Secondary Data Analyses (STROSA) [25, 26].

### Data sources

The data used for the study can be separated into information on a hospital and a patient level, respectively. Data on the patient level were derived from statutory health insurance data of eleven regional AOK insurances covering about 27 million patients in total, which is roughly a third of the German population. These data were provided by the AOK research institute (WIdO). The provided data include the years 2009–2017, as well as a pre-period of 3 years (2006–2008) for identification of incident cases. The data contain sociodemographic information such as birth/death dates and sex. The data source also covered information about in/outpatient diagnoses and procedures, medical prescriptions, as well as hospital admission/discharges based on widely used classification systems (ICD-10-GM, OPS, EBM, ATC). Note that the data contain no information about tumor stage and grade as is, e.g., the case in cancer registries, however, as stage IV in colon and rectal cancer is separated from stages I–III via distant metastasis, we use the presence of distant metastasis as a proxy for stage IV cancer. The study team is experienced in using administrative healthcare data for health services research studies and is considered the Good Practice Secondary Data Analysis from the German Society of Epidemiology [27].

Data on hospital certification was provided by the GCS, with information on the status of certification of hospitals and the associated time span. In addition, information on the hospital level was supplemented from the Standardized quality reports (SQR Standardized Quality Reports by the Federal Joint Committee, German: Standardisierte Qualitätsberichte des G-BA), which are compulsory for German hospitals and are available on a bi-annual basis for older and an annual basis for reports since 2013. To maintain a fixed interval between reports, we used reports from the years 2010, 2012, 2014, and 2016, with the years in between mapped to the most recent report. The SQR provides information on, e.g., the number of hospital beds, and hospital status (university/teaching hospital).

### Data protection and ethics

Data were pseudonymized using pseudonymization procedures at the patient and the hospital level, respectively. Pseudonymization on both levels was conducted by WIdO. The data was encrypted for transfer and subsequently analyzed at the Center for Evidence-based Healthcare (ZEGV) of the TU Dresden, Germany. The WiZen study was approved by the ethics committee of the TU Dresden (approval number: EK95022019, IRB 00001473, OHRP IORG0001076). Data processing and analyses were conducted in line with the Declaration of Helsinki and the General Data Protection Regulation of the European Union.

### Inclusion and exclusion criteria

The study population consists of incident patients with primary inpatient diagnosis of colon or rectal cancer in the period 2009–2017, continuously insured by the AOK until the end of the study period or death, whatever occurred first. Patients were required to be insured over the entire period of time (except for interruptions < 2 weeks) and had to be 18 years of age or older at the time of first cancer diagnosis. Upon the primary inpatient diagnosis, patients were required to have no inpatient diagnosis at least three years prior to diagnosis to ensure cases were incident. As an additional requirement to identify incident cases, patients were required to have no outpatient diagnosis of colon or rectal cancer of at least three years up to one year prior to diagnosis; the last year was excluded to account for preliminary examinations that precede hospitalization. Further reasons for exclusion were (i) treatment in a hospital that became a certified center within one year before diagnosis, (ii) primary resection > 6 months after primary diagnosis, (iii) survival time of zero days, and (iv) missing hospital characteristics. Note that hospital characteristics were not available for all hospitals as the underlying data stem from the SQR that tends to be incomplete. A detailed overview of the criteria and the underlying rationale can be found in Supplemental Text S1 and Table S1.

### Outcome

The primary outcome was the overall survival of incident patients diagnosed with colon and rectal cancer since index treatment. The index treatment was defined via the admission date of the first relevant entity-specific inpatient treatment, i.e., a treatment with primary diagnosis C18, C19 for colon, or C20 for rectal cancer. The index treatment thus corresponds to the first hospital stay due to colorectal cancer. Dates of death were considered up to December 31, 2017, and patients without a documented death date were treated as right-censored. Note that cancer registries typically use the date of histological examination for calculations of survival and in this way approximate the “onset of treatment”. As histological examination can not be reliably defined in billing data, we refer to the index treatment instead, i.e., the first intervention in the hospital setting.

### Treatment in certified cancer center

We separated our cohort into patients who received treatment in a certified colorectal cancer center and patients who have received treatment in another hospital as follows: A patient was considered as “treated in a certified cancer center” if the hospital where the first relevant treatment had taken place has been certified by the GCS prior to the admission date associated with that treatment. Patients treated in hospitals that became certified after this date count as “not treated in a certified center”. The first relevant treatment was defined as the resection, documented through the OPS codes associated with colon and rectal resection (Supplemental Table S1) in combination with the primary inpatient diagnosis C18, C19, or C20, respectively. In case of no resection, we referred to the first inpatient treatment with primary diagnosis C18, C19, or C20. If hospitals form an association in which at least one hospital is certified, our data does not allow for the unambiguous assignment of single hospitals with a certificate. We considered patients to be treated in a certified colorectal cancer center if at least one of the hospitals in an association is certified which results in a conservative estimate of the certification effect. In the remainder of the article, we refer to the patients who received treatment in a certified center as the “certified hospital group” and to patients who have received treatment in another/non-certified hospital as the “non-certified hospital group”. In both groups, follow-up starts with index treatment, i.e., at the admission date of the initial inpatient treatment.

### Covariates

Our study is retrospective and thus potentially subject to confounding. Therefore, we seek to minimize these biases by addressing a wide range of confounding variables for treatment in certified cancer centers both at the patient and the hospital level. These included, on the patient level, sex (male, female), age at index treatment, group 18–59, 60–79, and 80 + , distant metastasis as a proxy for stage IV cancer prior to or upon first diagnosis of colon or rectal cancer (ICD: C78-C79), other oncological diseases (ICD: all of C except for C18/C19 or C20 and C77-C79, labeled as “secondary malignoma” in the analysis) and Elixhauser comorbidities [28]. These comorbidities consist of groups of ICD codes and have been defined such as to account for an increased risk of hospital mortality. We use the version adapted to ICD-10 by Quan [29] and restrict the groups to those relevant for colon and rectal cancer as selected by clinical experts. These comorbidities included congestive heart failure, cardiac arrhythmias, valvular disease, pulmonary circulation disorders, peripheral vascular disorders, hypertension (complicated/uncomplicated), other neurological disorders, chronic pulmonary disease, diabetes (complicated/uncomplicated), renal failure, liver disease, deficiency anemia, alcohol abuse, and drug abuse. On the hospital level, we adjusted for the number of hospital beds, grouped 1–299, 300–499, 500–999, and 1000 + , and for hospital status (teaching hospital, university hospital, and ownership). Finally, we adjusted for the calendar year of index treatment to take into account the effects of medical progress, incomplete washout at the beginning, and more frequent censoring at the end of the observation period with the aim of capturing a potential overestimate of the certification effect.

### Statistical methods

All statistical procedures were fixed in a detailed statistical analysis plan prior to data access. We used descriptive statistics to characterize the cohort patient and hospital characteristics in (i) total, the (ii) certified hospital, and (iii) non-certified hospital group, through median and quartiles (Q1;Q3) if variables were continuous, and in absolute numbers and percentages if variables were categorical. We estimated the overall and relative survival stratified by center status. To appropriately address excess mortality due to colon and rectal cancer in both groups, we used age/sex-specific mortality data of the German population for relative survival estimation [30]. Relative survival, in contrast to overall survival, separates death by mortality based on age and sex in the (German) population as published by the Federal Office of Statistics [31] from disease-specific mortality, in this case, cancer, see [32] for the implementation in R and [33] for a general introduction. To adjust for confounding, we used a Cox model with shared frailty [34, 35]. This class of models enables adjustment on different levels (patient, hospital) as (i) correlations between outcomes for patients treated in the same hospital are accounted for and (ii) unknown hospital characteristics that are independent of the known confounders are adjusted for. We compared the results of Cox regression models for different sets of confounders. We started from a core set (age, sex) that was, in the next step, increasingly supplemented by confounders on the patient (metastases, secondary malignoma) and, in a third step, on the hospital level. Finally, we included dummy years in the full model to account for time-dependent effects on survival such as a potential improvement in therapy. In a series of sensitivity analyses, we stratified for different covariates including those that were distributed differently between groups to check for effect modification. The covariates used in sensitivity analyses were sex, secondary malignoma, distant metastasis, and hospital association. Finally, we re-run the analysis upon the introduction of a variable indicating the duration of certification.

## Results

### Inclusion and exclusion

**Fig. 1:**
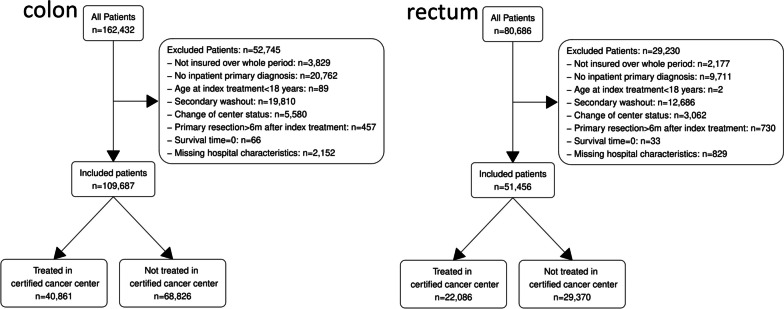
Flow chart showing inclusion and exclusion of patients for colon and rectal cancer

Figure 1A shows a flow chart with the inclusion and exclusion of patients along with the reasons for exclusion for colon cancer. 109,520 patients out of a total of 162,432 patients diagnosed as pre-selected by the data site (see Supplemental information S1 for selection criteria), were included in the cohort. 40,748 patients were treated in a certified cancer center, while 68,772 patients were treated in a hospital without certification. For rectal cancer, 51,417 pre-selected patients were included out of a total of 80,686 patients, and 22,045 patients were treated in a certified cancer center, while 29,372 patients were treated in a non-certified hospital (Fig. 1B). As colon and rectal cancer can sometimes co-exist, there was an overlap of 4394 patients in the cohorts for colon and rectal cancer, which corresponds to 4.0% of patients diagnosed with colon and 8.5% of patients diagnosed with rectal cancer.

### Patient and hospital characteristics

**Fig. 2:**
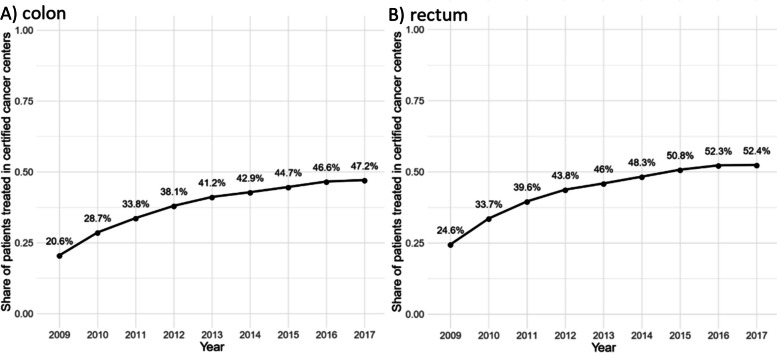
Share of included patients treated in certified cancer centers for colon (**A**) and rectal (**B**) cancer, 2009–2017

Table 1 shows baseline characteristics for colon (A) and rectal cancer (B) for all patients, and stratified by treatment in certified centers and non-certified hospitals. In the case of colon cancer (Table 1), the characteristics did not differ considerably for patients with treatment in certified centers and non-certified hospitals: the median age upon diagnosis was 75 years. Roughly 50% of patients were male, and about 27% had distant metastases. Rectal cancer (Table 1) was more prominent in men (60%), the median age was 73 years, and about 26% of patients had distant metastasis. Again, characteristics were almost equally distributed in certified centers and non-certified hospitals, with the exception of non-certified hospitals treating a few percentage points more patients in the age group 80 + , a covariate with great influence on survival prospects (33.4% vs. 30.5% for colon, 26.9% vs. 21.8% for rectum). The most prominent comorbidities in both cohorts were hypertension (uncomplicated, 82%/77%), chronic pulmonary disease (43%/40%), diabetes (uncomplicated 39% / 36%), congestive heart failure (37%/31%), cardiac arrhythmia (33%/38%), peripheral vascular disorders (30%/31%) for colon and rectal cancer, respectively. For both cohorts, comorbidities were distributed similarly in certified centers/non-certified hospitals. Note that Clopper-Pearson confidence intervals for both groups do not overlap in most cases due to the large sample size (Figure S2a, b). For both colon and rectal cancer, the fraction of patients treated in certified centers had increased over time from roughly 20% of patients in 2009 to approximately 50% in 2017 (Fig. 2).

**Table 1:** Patient characteristics for colon (A) and rectal cancer (B)

Variable	All, *n*	All, %	Not certified, *n*	Not certified, %	Certified, *n*	Certified, %
(A)						
Age in years, median (Q1;Q3)	75		75		75	
Age in years, (Q1;Q3)	(67;81)		(68;82)		(67;81)	
Age 18–59	13,177	12%	7969	11.6%	5208	12.7%
Age 60–79	61,050	55.7%	37,863	55%	23,187	56.7%
Age 80 +	35,460	32.3%	22,994	33.4%	12,466	30.5%
Sex female	54,753	49.9%	34,753	50.5%	20,000	48.9%
Sex male	54,934	50.1%	34,073	49.5%	20,861	51.1%
Distant metastasis yes	29,957	27.3%	18,204	26.4%	11,753	28.8%
Other oncological disease	39,926	36.4%	24,653	35.8%	15,273	37.4%
Congestive heart failure	40,841	37.2%	26,392	38.3%	14,449	35.4%
Cardiac arrhythmias	42,028	38.3%	26,202	38.1%	15,826	38.7%
Valvular disease	21,941	20%	13,578	19.7%	8363	20.5%
Pulmonary circulation disorders	9052	8.3%	5554	8.1%	3498	8.6%
Periph. vascular disorders	33,766	30.8%	21,008	30.5%	12,758	31.2%
Hypertension (uncomplicated)	90,269	82.3%	56,901	82.7%	33,368	81.7%
Hypertension (complicated)	30,711	28%	19,429	28.2%	11,282	27.6%
Other neurological disorders	12,472	11.4%	7808	11.3%	4664	11.4%
Chronic pulmonary disease	47,559	43.4%	29,258	42.5%	18,301	44.8%
Diabetes (uncomplicated)	42,842	39.1%	26,988	39.2%	15,854	38.8%
Diabetes (complicated)	23,684	21.6%	14,736	21.4%	8,948	21.9%
Renal failure	27,569	25.1%	17,172	24.9%	10,397	25.4%
Liver disease	30,531	27.8%	18,414	26.8%	12,117	29.7%
Blood loss anemia	10,219	9.3%	6512	9.5%	3707	9.1%
Deficiency anemia	29,786	27.2%	18,403	26.7%	11,383	27.9%
Alcohol abuse	7751	7.1%	4,805	7%	2946	7.2%
Drug abuse	2644	2.4%	1720	2.5%	924	2.3%
Total	109,687		68,826		40,861	
(B)						
Age in years, median (Q1;Q3)	73		73		72	
Age in years, (Q1;Q3)	(64;79)		(65;80)		(63;79)	
Age 18–59	8560	16.6%	4,410	15%	4150	18.8%
Age 60–79	30,173	58.6%	17,056	58.1%	13,117	59.4%
Age 80 +	12,723	24.7%	7,904	26.9%	4819	21.8%
Sex female	20,621	40.1%	11,990	40.8%	8631	39.1%
Sex male	30,835	59.9%	17,380	59.2%	13,455	60.9%
Distant metastasis	13,162	25.6%	7,520	25.6%	5642	25.5%
Other oncological disease	25,631	49.8%	14,589	49.7%	11,042	50%
Congestive heart failure	15,671	30.5%	9,566	32.6%	6105	27.6%
Cardiac arrhythmias	16,898	32.8%	9,733	33.1%	7165	32.4%
Valvular disease	8439	16.4%	4,878	16.6%	3561	16.1%
Pulmonary circulation disorders	3138	6.1%	1,832	6.2%	1306	5.9%
Periph. vascular disorders	15,565	30.2%	8,870	30.2%	6695	30.3%
Hypertension (uncomplicated)	39,799	77.3%	23,062	78.5%	16,737	75.8%
Hypertension (complicated)	12,267	23.8%	7,254	24.7%	5013	22.7%
Other neurological disorders	5061	9.8%	2,983	10.2%	2078	9.4%
Chronic pulmonary disease	20,446	39.7%	11,417	38.9%	9029	40.9%
Diabetes (uncomplicated)	18,256	35.5%	10,651	36.3%	7605	34.4%
Diabetes (complicated)	9711	18.9%	5578	19%	4133	18.7%
Renal failure	10,487	20.4%	6094	20.7%	4393	19.9%
Liver disease	13,441	26.1%	7308	24.9%	6133	27.8%
Blood loss anemia	2682	5.2%	1643	5.6%	1039	4.7%
Deficiency anemia	7977	15.5%	4631	15.8%	3346	15.1%
Alcohol abuse	4530	8.8%	2572	8.8%	1958	8.9%
Drug abuse	1099	2.1%	654	2.2%	445	2%
total	51,456		29,370		22,086

Table 2 shows characteristic variables on the hospital level for all hospitals that have treated patients with colon cancer, certified centers, and hospitals without certification. As certificates were issued jointly for colon and rectal cancer, these characteristics differ by less than 2% for both colon (C18/19) and rectal cancer (C20); the data for rectal cancer can be found in Table S3. Hospitals holding certificates tended to have a higher number of beds: 40 out of 481 hospitals (8%) with less than 300 beds held a certificate, whereas 41 out of 50 hospitals (82%) with 1000 + beds did. Teaching and university hospitals were more likely to be certified: note that these hospitals also tend to be large. There was a slight tendency of public hospitals to hold a certificate as compared to non-profit/private hospitals.

**Table 2:** Baseline table for hospital characteristics for patients with colon cancer

Variable	All	*n* = 1088	Certified: no	*n* = 777	Certified: yes	*n* = 311
Hospital beds, *n* (%)						
1–299	614	56.4%	574	73.9%	40	12.9%
300–499	262	24.1%	142	18.3%	120	38.6%
500–999	162	14.9%	52	6.7%	110	35.4%
1000 +	50	4.6%	9	1.2%	41	13.2%
Teaching hospital, *n* (%)						
No	469	43.1%	419	53.9%	50	16.1%
Yes	619	56.9%	358	46.1%	261	83.9%
University hospital, *n* (%)						
No	1059	97.3%	769	99%	290	93.2%
Yes	29	2.7%	8	1%	21	6.8%
Hospital ownership, *n* (%)						
Public	388	35.7%	245	31.5%	143	46%
Non-profit	473	43.5%	350	45%	123	39.5%
Private	227	20.9%	182	23.4%	45	14.5%
Certified center, *n* (%)						
No	777	71.4%				
Yes	311	28.6%				

#### Survival

**Fig. 3:**
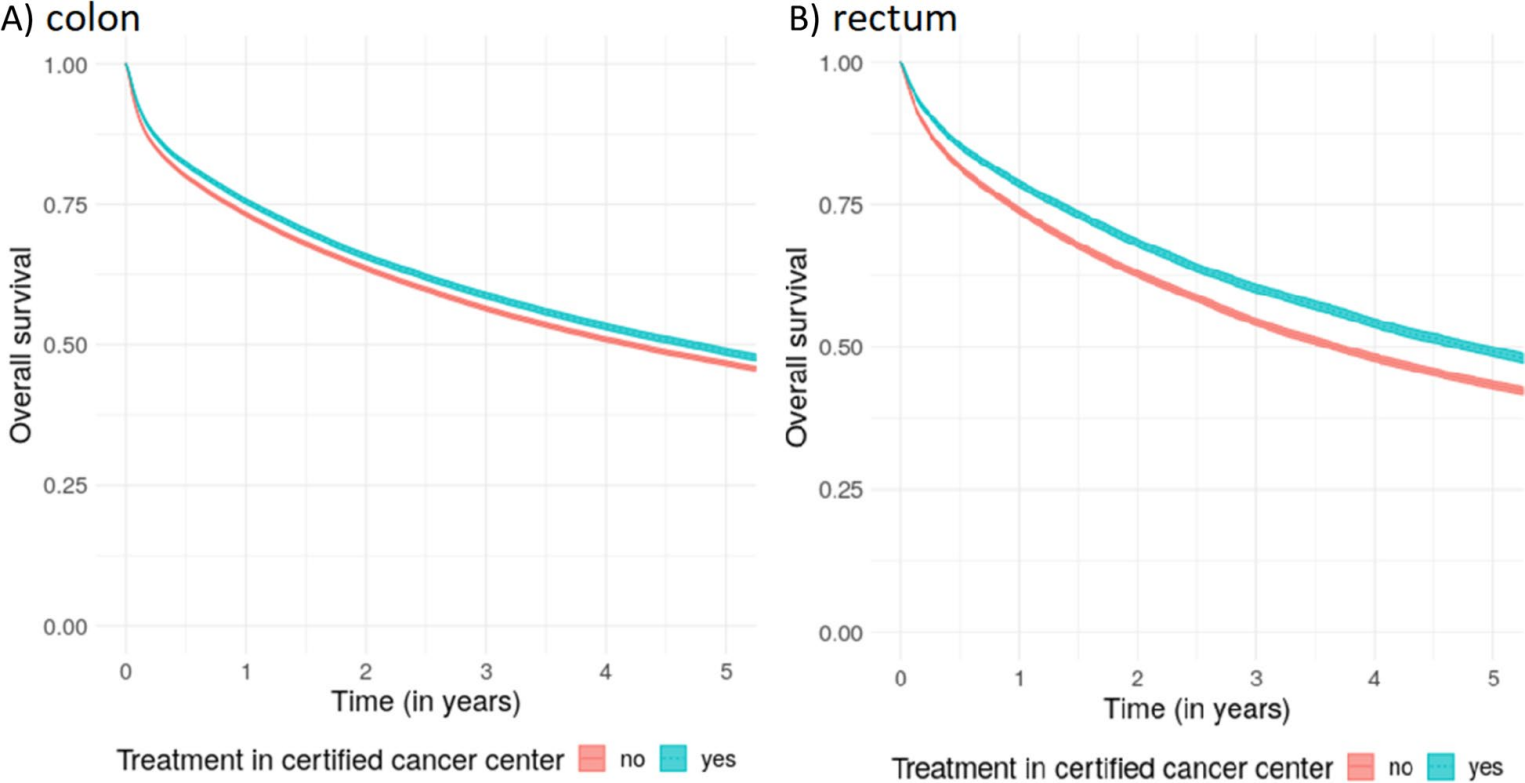
Overall survival with 95% confidence intervals by center status for colon (**A**) and rectal cancer (**B**)

**Fig. 4:**
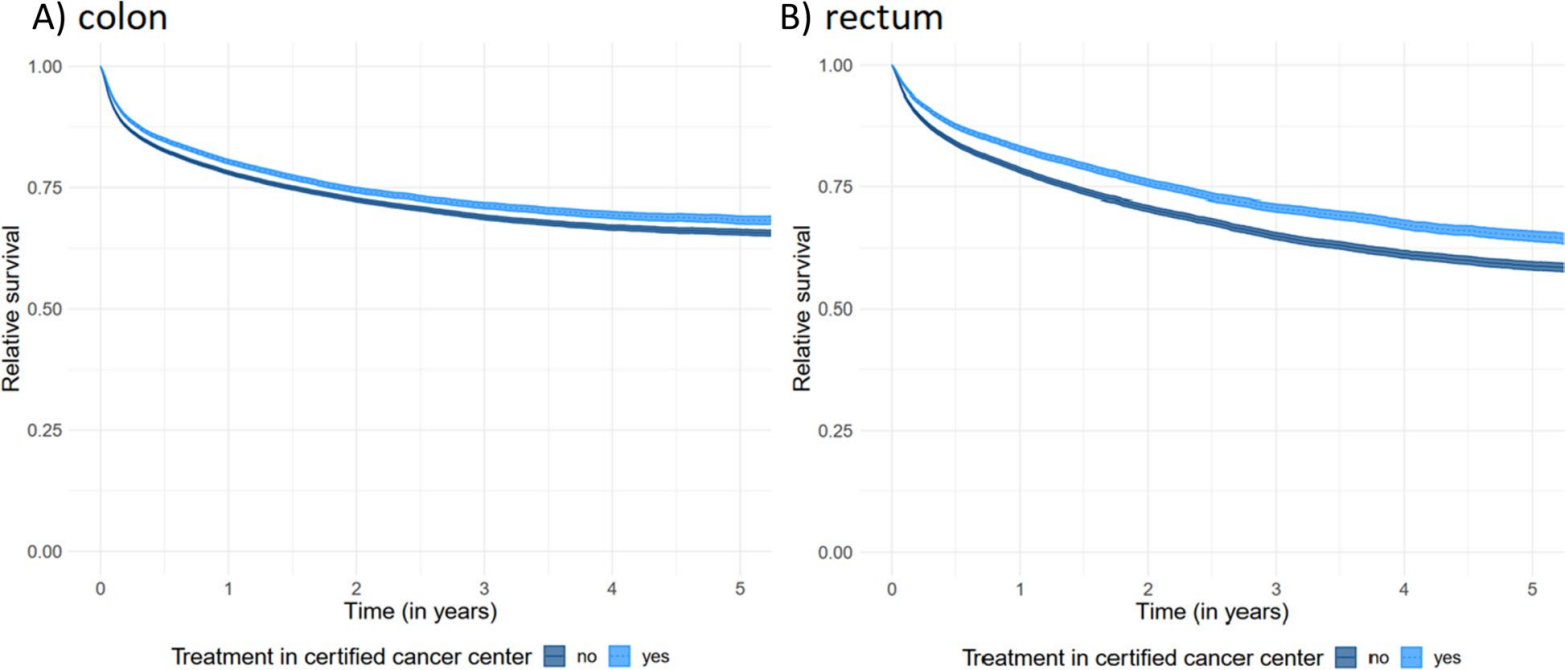
Relative survival with 95%-confidence intervals by center status for colon (**A**) and rectal cancer (**B**)

The *overall* survival for patients diagnosed with colon and rectal cancer stratified by treatment in certified centers and non-certified hospitals with 95% confidence bands is shown in Fig. 3A and B and Table S4. In both entities, patients treated in certified centers had a significantly better chance of survival compared to patients treated in non-certified hospitals (non-overlapping CIs). This effect remained stable for the corresponding *relative* survival shown in Fig. 4. The survival effect was more prominent for rectal cancer: Relative survival after 1 year was 82.9% with CI = 82.3–83.5% in certified centers and 78.4% with CI = 77.9–78.9% in hospitals without certification for rectal cancer, respectively. After 5 years, survival was 65.0% (CI = 63.9–66.0%) in certified centers vs 58.8% (CI = 57.9–59.6%) in non-certified hospitals, respectively. For colon cancer, 1- and 5-year relative survival was also significantly better in certified centers compared to non-certified hospitals, with 80.4% (CI = 79.9–80.8%) in certified centers compared to 78.1% (CI = 77.7–78.5%) in non-certified hospitals after one and 68.3% (CI = 67.5–69.2%) vs. 65.8% (CI = 65.2–66.4%) after 5 years. The advantage in relative survival also held true for both colon and rectal cancer upon stratification of hospital beds into the four categories defined previously (0–299, 300–499, 500–999, 1000 + beds) (see Figure S1).

### Main regression results

We present estimates of the center effect by computing the Hazard Ratio (HR) for different sets of counfounders. The results of Cox regression with shared frailty are summarized in Table 3, and full results can be found in Tables S5 and S6. As indicated by the survival analysis, the raw estimate of the center effect pointed to better survival of patients treated in certified centers, with the effect being more pronounced for rectal (HR = 0.86, *p* < 0.001) compared to colon (HR 0.93, *p* < 0.001) cancer (Table 3). After adding sociodemographic characteristics, the beneficial effect of being treated in a certified hospital remained unchanged for colon cancer and increased slightly, but not significantly, for rectal cancer. For both types of cancer, survival prospects became worse with increasing age: for patients 60–79 years of age, HR from the model that took solely certification, age, and sex into account, was, for colon cancer (HR = 1.73, *p* < 0.001) and rectal cancer with HR = 1.76, *p* < 0.001. For patients of age 80 + , the results are HR = 3.58, *p* < 0.001 (colon) HR = 3.79, *p* < 0.001 (rectum). Both values are with respect to the reference group 18–59 years. Survival prospects were worse for male compared to female patients with a HR = 1.17, *p* = 0.001 for colon and HR = 1.11, *p* < 0.001 for rectal cancer.

**Table 3:** Hazard ratios (HR) with 95% confidence intervals (CI) from Cox regressions with shared frailty

	Colon		Rectum	
ref: no	HR	CI	HR	CI
Certification only	0.93***	(0.90,0.96)	0.86***	(0.83,0.89)
Certification, age, sex	0.94***	(0.91,0.96)	0.88***	(0.86,0.91)
Certification, age, sex, metastases, secondary malignoma, comorbidities	0.89***	(0.87,0.92)	0.88***	(0.85,0.90)
Certification, age, sex, metastases, secondary malignoma, comorbidities, hospital characteristics	0.88***	(0.86,0.91)	0.88***	(0.85,0.92)
Certification, age, sex, metastases, secondary malignoma, comorbidities, hospital characteristics, calendar year dummies	0.92***	(0.89,0.95)	0.90***	(0.87,0.94)

*HR* hazard ratio, *CI* 95% confidence interval, significance levels: *5%, **1%, ***0.1%

The model that took into account certification, age, sex, secondary malignoma, and Elixhauser comorbidities showed that the presence of distant metastasis had a considerable impact on survival prospects, with HR = 4.19, *p* < 0.001 for colon and 3.59, *p* < 0.001 for rectal cancer. The Elixhauser comorbidities that had a negative influence on survival (HR > 1.2) were, for both colon and rectal cancer, pulmonary circulation disorders, other neurological diseases, alcohol and drug abuse, and renal failure (see Tables S5 and S6). Adding hospital characteristics and calendar dummies (i.e., the year of diagnosis as a categorical variable) did not considerably alter the results for colon cancer (HR = 0.92, *p* < 0.001) but reduced the estimated effect of certification for rectal cancer, resulting in HR = 0.90 (*p* < 0.001) for the full set of confounders. There was no evidence for better survival associated with any hospital characteristics. This also holds true when running models consisting of the certification and one hospital characteristic only (see Table S7). Survival prospects were influenced by calendar year for both types of cancer, with a steady reduction of HR over the years, which became significant from 2012 (2014) for colon (rectal) cancer and ended up at HR = 0.82 for colon and 0.83 for rectal cancer 2017 with respect to the reference year 2009. Including calendar years also slightly narrowed the HR for certification, albeit within confidence intervals. The results for the full model remained stable for a standard Cox regression without frailty, with a slightly lower HR for the certification (HR = 0.90, *p* < 0.001) for colon cancer, and unaltered HR (HR = 0.90, *p* < 0.001) for rectal cancer (Table S7). Concordance (Harrel’s *C*) increased with model complexity, ranging from 0.57 to 0.74 for colon and 0.58 to 0.73 for rectal cancer, with the most prominent increase taking place upon the addition of Elixhauser comorbidities (S5, S6). In conclusion, after adjustment for relevant confounders, the estimated fully adjusted effect indicated an 8% and 10% better survival for patients suffering from colon or rectal cancer who have been treated in a certified center compared to those who have not.

### Sensitivity analysis

The beneficial effect on survival prospects in the certified hospital group remained robust against stratification for sex, secondary malignoma, distant metastasis, and single hospital/association in both colon and rectal cancer using Cox regression with shared frailty (Supplemental tables S8, S9). In all of the subgroups, the influence of the calendar year on survival prospects remained robust as well. We further inspected the time spans between the first documentation of cancer, which is likely to correspond to the onset of diagnostic measures that may take place either in the in- or outpatient sector, and the date of index treatment: The time span between “date of index treatment”, i.e., the beginning of the first hospital stay due to colorectal cancer, and the “date of first in OR outpatient C- diagnosis” was less than 4 weeks for 80–90% of patients across groups and entities, and most patients received surgery at the day or within 4 weeks of index treatment (Table S10). The benefit in survival remained for the subgroup of “resected only “ patients, and upon using the „date of first diagnosis “ as a starting point for survival time (Table S11). Hence, the “date of index treatment” provided a robust time point for survival analysis. In a final sensitivity analysis, we replaced the binary separation of certified hospital and non-certified hospital group with a variable indicating the duration of certification into the 4 categories certified < 1 year, 1– < 2 years, 2– < 5 years, and 5 or more years (Table S12). For both colon and rectal cancer, there was a beneficial effect of certification, which increased with its duration.

## Discussion

This large nationwide cohort study extends previous research on the effects of certification of hospitals for evidence-based standards for the treatment of patients with colorectal cancer in various important aspects. Internationally, it has been shown that an NCI designation is associated with a lower risk of postoperative death and improved long-term survival for colon and rectal cancer [16]. While previous studies covering the GCS program [11, 12, 21] were based on regional samples and shorter observation periods, our study was based on a large nationwide cohort of more than 150,000 patients with incident colon or rectal cancer. Our study further covered more than one thousand hospitals and a time span of more than a decade. We took important covariates such as comorbidities (patient level) and hospital size (hospital level) into account, thereby overcoming some of the limitations of previous studies. Our results agree with analyses based on cancer registry data that have also been conducted as part of the WiZen project, covering the registries Dresden, Erfurt, Regensburg, and Berlin-Brandenburg [36, 37]. These data contain tumor-specific information such as staging and grading, and the survival analyses also show a beneficial effect of certification [38]. In those data, the separation of stages I–III from stages IV is used in sensitivity analyses and shows that the certification effect is stronger for patients in stage I–III for both colon and rectal cancer, in agreement with our results for colon but not rectal cancer (S8, S9).

Our study has important implications for clinical care and health policy, as it shows robust evidence in favor of treatment in certified cancer centers compared to non-certified hospitals. This is particularly important in light of the fact that only a minority of patients were actually treated in certified hospitals.

The advantage in survival for patients who have been treated in certified centers yielded 8% and was stable across a wide variety of sets of confounding variables, thus controlling for many relevant patient and hospital characteristics. Sensitivity analyses further underlined the robustness of this result. Hospital characteristics were distributed differently across certified hospital and non-certified hospital groups and were adjusted for in regression analyses. We did not find any evidence for an influence of hospital characteristics using a rather broad categorization. The connection between hospital characteristics such as hospital size, and more specific characterization such as equipment, specialization, and survival has been discussed in the USA on a broad level [39], and also for cancer [40]. Other examples also covered in Europe are cardiac arrest and stroke [41–44], often finding that measures targeted at a specific condition improve survival. Another important result is that on the patient level, the distribution of patients’ comorbidities was similar in both groups (certified/non-certified). We did not find hints of a centralization effect, i.e., that sicker patients would be more likely to be sent to a center. As hospitals that belong to an association were treated as certified, the benefit in survival might even be larger than estimated. An interesting issue would be to identify the subgroup of very frail, care-dependent patients in the age group 80 + which differed in certified/non-certified group through, e.g., pharmaceutical treatment such as antidementive drugs, However, the definition of drug administration in billing data is an issue too complex to be covered within the scope of this paper. Finally, the decrease in HR for certification upon the addition of calendar years into the model might reflect that medical progress might not fully be captured by that variable alone. Our findings thus support the hypothesis that patients who have been treated in certified centers have better survival outcomes than patients who have been treated in non-certified hospitals. Through consideration of a broad and generalized set of variables for confounding we intended to minimize risk of bias and enable comparability of the certification effect across different types of cancer. The survival curves for both colon and rectal cancer separate within the first year, suggesting that quality, and safety of surgery may have an effect.

Previous analyses on the benefits of survival prospects for patients with colon cancer who have received treatment in a certified center have been restricted to several thousand patients from specific regions of Germany [11, 12], and evidence for survival prospects for patients with rectal cancer stems from a single center [21]. Through evidence from a large nationwide cohort, the rather fragmented evidence on the survival benefits of treatment in certified cancer centers is strengthened substantially. As the GCS certification program puts a strong emphasis on the implementation of standardization [17], our evidence provides a good starting point for conducting similar studies in non-German ECC-certified centers.

We were able to show that an 8% improvement in overall survival for patients with colon cancer and even 10% for patients with rectal cancer could be achieved by certifying a hospital. This is particularly interesting, as a clear survival benefit for patients could be achieved by means, e.g., steering patients into CRC-certified centers, and, as these structures are already established, with little or no additional costs for the health care system. An improvement in survival prospects has already been demonstrated in NCI-designated cancer centers [45, 46]. Furthermore, in Germany, it has been shown that high-volume hospitals could significantly reduce in-hospital mortality [47]. In addition, international studies have shown that not only caseload but also surgical quality, which is also emphasized in certification programs, improves outcomes [48, 49]. If one puts the magnitude of the survival benefit from certification into relation to current oncological studies that have received approval in colorectal cancer therapy, the effect shows to be of particular relevance: in the CORRECT Study Group that includes patients with metastatic colorectal cancer, the median overall survival was significantly prolonged from 5.0 to 6.4 months with the administration of regorafenib [50]. TAS-102 was able to prolong overall survival from 5.3 to 7.1 months [51]. Both studies led to the approval of the drugs in CRC therapy. In comparison, the effect on overall survival through the administration of new drugs improved survival, but with significantly higher costs for the health care system than could be achieved by centralization in certified colorectal cancer centers. C. Cheng et al. [52] were also able to show that treatment in certified centers is more cost-effective in addition to the better overall survival of the patients. To summarize, the results presented in this study speak in favor of centralizing treatment in certified centers for CRC.

### Strengths and limitations

Our study has several important strengths: it has a low degree of selectivity as the documentation of primary diagnoses and treatments is subject to legal regulations for billing [53]. Hence, the variables in question can be regarded as complete, making survival a particularly suitable outcome. A large set of patient-specific confounders, such as comorbidities, could be included. Additional entity-specific adjustments to the confounders as well as inclusion and exclusion criteria would have the potential to further sharpen the results, but could not be implemented due to the necessary uniformity and comparability of the analyses across all entities considered in the study. Due to the observational nature of our study, causal conclusions may not be drawn, even though the robustness of our findings strongly suggests that they are valid. As the status “certification” encompasses a complex structure of interventions that concern the entire treatment on the hospital level that is hard to quantify, most, if not all, studies targeted at the evaluation of certification are subject to this limitation. The specific treatment, emergency management, and post-operative morbidity influence patient survival. However, to avoid mediation bias [54], these were not allowed to be included in the adjustment. Health insurance data cover a limited time span, such that incident cases have to be estimated from the data. On the (general) patient level, there is no information about socioeconomic status. The data also do not allow for the separation of patients’ or hospital locations into rural and urban areas due to restrictions on data privacy protection. However, recent literature suggests that accessibility of inpatient care is sufficiently high in Germany [55] such that regional aspects might be secondary to a serious disease such as cancer. As our observation period ends in the year 2017, scientific progress that has been implemented in cancer care and for recent years is not covered by the cohort.

Another limitation of health insurance data is that important cancer-related confounders, such as tumor stage, histology, or grading of the tumor are not part of those data. Note that we seek to overcome this limitation within the WiZen project by means of linking data from four cancer registries with the administrative data in accordance with the Good Practice of Secondary Data Analysis [26]. Within the data discussed in this paper, distant metastasis was used as a proxy for high tumor stages. As our data come from a single health insurance, it was not possible to include the total patient volume in the analysis. Volume has been shown to influence relevant outcomes such as survival [56–58]. As GCS certification does require a minimal volume, part of our findings may thus be due to the effects of volume. However, we currently are not aware of a feasible way to quantify caseload due to the lack of data availability except for access to complete national data or data that allow a definition of caseload within standardized quality reports [59, 60].

## Conclusions

To exploit the advantages of the data (large sample size, nationwide, almost non-selective cohort), we focused on a large set of patient and hospital characteristics that describe the cohort in a rather generalized way. Adjustment for these characteristics in a series of increasingly complex models indicated the robustness of our overall finding that certification does have a stable, positive impact on survival for both colon and rectal cancer. It would be desirable to analyze further aspects of certification such as guideline adherence, e.g., definitive surgery + adjuvant chemotherapy for patients with stage III colon cancer. Although we have information that can serve as a proxy for specific therapies in the data, such as resection, chemotherapy, radiation treatments, and palliative care, those variables can not be used in a meaningful way without knowledge of the tumor stage. This highlights the importance of analysis of linked data sets such as registry (that contain staging and grading information) and billing data (that contain reliable information about therapies), as these contain the desired information.

An in-depth analysis, ideally in the form of target trial emulation which provides a framework for causal inference from observational data [61, 62] of whether there is an overall difference in specific therapeutic measures taken to treat colorectal cancer in certified centers vs. non-certified hospitals thus remains a subject for further investigation.

The presented study contributes stable evidence about benefits in survival for patients with colorectal cancer who have been treated in a GCS-certified cancer center. These benefits may partly be due to volume [47] and quality of surgery [48, 49], but also adherence to quality standards such as guideline adherence in diagnostic procedures and therapy [63–65], i.e., a coordinated effort to conduct this complex intervention. In summary, this study provides robust evidence that patients with incident colon or rectal cancer most likely benefit from treatment in a certified hospital. This important information should be widely distributed to patients, referring outpatient physicians, and decision-makers in the healthcare system and health policy.

### Supplementary Information

**Additional file 1:** **Supplemental text S1.** Exclusion criteria and primary selection. **Table S1. **OPS-codes for definition of resection (in German). **Figure S1.** Relative survival stratified by hospital size. **Table S2a.** Clopper-Pearson Intervals of baseline table for patients with colon cancer by certification status with interval overlap. **Table S2b.** Clopper-Pearson Intervals of baseline table for patients with rectal cancer by certification status with interval overlap. **Table S3.** Baseline table for hospital characteristics for patients with rectal cancer by certification status. **Table S4. **Overall survival rates for 30days, 1, 2, 3, 4, 5 years with 95% CI-intervals (lower, upper). **Supplemental Table S5.** Full results of Cox regression with shared frailty for colon cancer. **Supplemental Table S6.** Full results of Cox regression with shared frailty for rectal cancer. **Table S7.** Hazard ratios (HR) with 95%-confidence intervals (CI) from Cox regressions with shared frailty for certification effect and each hospital characteristic separately (1a-d), and a full model with standard Cox regression (1f).**Tables S8. **Colon cancer: Hazard ratios (HR) with 95%-confidence intervals (CI) from Cox regressions with shared frailty for different subgroups of patients and hospitals. **Table S9.** Rectal cancer: Hazard ratios (HR) with 95%-confidence intervals (CI) from Cox regressions with shared frailty for different subgroups of patients and hospitals. **Table S10. **Descriptive statistics of subgroups of patients with resection date/date of first diagnosis within a given time span from date of index treatment. **Table S11. **Hazard ratios (HR) with 95%-confidence intervals (CI) from Cox regressions with shared frailty for resection/no resection and analysis with “date of first diagnosis” instead of “date of index treatment”. **Table S12. **Hazard ratios (HR) with 95%-confidence intervals (CI) from Cox regressions with shared frailty including continuity of certification.

## Data Availability

The authors confirm that the data utilized in this study cannot be made available in the manuscript, the supplemental files, or in a public repository due to German data protection laws (‘Bundesdatenschutzgesetz’, BDSG). Therefore, they are stored on a secure drive in the AOK Research Institute (WIdO), to facilitate replication of the results. Generally, access to data of statutory health insurance funds for research purposes is possible only under the conditions defined in German Social Law (SGB V § 287). Requests for data access can be sent as a formal proposal specifying the recipient and purpose of the data transfer to the appropriate data protection agency. Access to the data used in this study can only be provided to external parties under the conditions of the cooperation contract of this research project and after written approval by the sickness fund. For assistance in obtaining access to the data, please contact wido@wido.bv.aok.de.

## Abbreviations

ATC: Anatomical therapeutic chemical classification system
AOK: Allgemeine Ortskrankenkasse
BDSG: Bundesdatenschutzgesetz/Federal law for privacy protection
BMBF: Bundesministerium für Bildung und Forschung/Federal ministry of education and research
BMG: Bundesministerium für Gesundheit/Federal ministry of health
CI: Confidence interval
CRC: Colorectal cancer
DGAV: Deutsche Gesellschaft für Allgemein- und Viszeralchirurgie e.V.
DGHO: Deutsche Gesellschaft für Hämatologie und Medizinische Onkologie e. V.
DKG: Deutsche Krebsgesellschaft/German cancer society
EBM: Einheitlicher Bewertungsmaßstab/German outpatient compensation system
EU: European Union
G-BA: Gemeinsamer Bundesausschuss/German federal joint committee
GCS: German cancer society, see: DKG
HR: Hazard ratio
ICD-10 (-GM): International statistical classification of diseases and related health problems, German modification
IQTIG: Institut für Qualitätssicherung und Transparenz im Gesundheitswesen/Institute for quality assurance and transparency in healthcare
NCI: National Cancer Institute
OPS: Adaptation of the international classification of procedures in medicine of the world health organisation
Q1/Q3: First and third quartile
SGB: Sozialgesetzbuch/German social law
SQR/SQB: Standardized quality reports/Standardisierte Qualitätsberichte der Krankenhäuser
TAS-102: Trifluridin/Tipiracil
TU Dresden: Technische Universität Dresden
UICC: Union for International Cancer Control
WIdO: Wissenschaftliches Institut der AOK (AOK research Institute)
WiZen: Wirksamkeit der Versorgung in onkologischen Zentren/Effectiveness of care in oncological centres
ZEGV: Zentrum für Evidenzbasierte Gesundheitsversorgung/Center for evidence-based healthcare
