# Supplementary material for: Treatment in certified cancer centers is related to better survival in patients with colon and rectal cancer: evidence from a large German cohort study
Source: World J Surg Oncol. 2024 Jan 6;22:11. doi: 10.1186/s12957-023-03262-9 (PMC10770882; doi:10.1186/s12957-023-03262-9)
Supplement: Supplementary file 1 — Additional file 1: Supplemental text S1. Exclusion criteria and primary selection. Table S1. OPS-codes for definition of resection (in German). Figure S1. Relative survival stratified by hospital size. Table S2a. Clopper-Pearson Intervals of baseline table for patients with colon cancer by certification status with interval overlap. Table S2b. Clopper-Pearson Intervals of baseline table for patients with rectal cancer by certification status with interval overlap. Table S3. Baseline table for hospital characteristics for patients with rectal cancer by certification status. Table S4. Overall survival rates for 30days, 1, 2, 3, 4, 5 years with 95% CI-intervals (lower, upper). Supplemental Table S5. Full results of Cox regression with shared frailty for colon cancer. Supplemental Table S6. Full results of Cox regression with shared frailty for rectal cancer. Table S7. Hazard ratios (HR) with 95%-confidence intervals (CI) from Cox regressions with shared frailty for certification effect and each hospital characteristic separately (1a-d), and a full model with standard Cox regression (1f).Tables S8. Colon cancer: Hazard ratios (HR) with 95%-confidence intervals (CI) from Cox regressions with shared frailty for different subgroups of patients and hospitals. Table S9. Rectal cancer: Hazard ratios (HR) with 95%-confidence intervals (CI) from Cox regressions with shared frailty for different subgroups of patients and hospitals. Table S10. Descriptive statistics of subgroups of patients with resection date/date of first diagnosis within a given time span from date of index treatment. Table S11. Hazard ratios (HR) with 95%-confidence intervals (CI) from Cox regressions with shared frailty for resection/no resection and analysis with “date of first diagnosis” instead of “date of index treatment”. Table S12. Hazard ratios (HR) with 95%-confidence intervals (CI) from Cox regressions with shared frailty including continuity of certification. [file 12957_2023_3262_MOESM1_ESM.docx]

## Supplementary material

## Supplemental text S1

## Exclusion criteria and primary selection

The definition for the primary selection at WIdO was as follows: Continuously insured (>350 days/year) with the AOK with german postal code (0< plz < 99999) in the years 2006-2017 or until death (**totalling ca**. **15,56 mio**). Incident diagnosis of colon (C18,C19) or rectal cancer with first inpatient diagnosis with hospital admission date within the years 2009-2017 with primary or secondary diagnosis C18/19 or C20, respectively.

The following sequential exclusion criteria (with rationales) were used for this pre-selected data set:

1. Not insured for the whole period (Relevant diagnoses, treatments, and events (incl. death) may not be observed), except for breaks up to 14 days or death. It is assumed that this exclusion does not introduce a sort of bias as changing the type of health insurance may have economic reasons, but is most likely independent of the type of treatment offered healthcare, i.e. we treated the scenario of “having more than one insurance throughout the observation period” as equal with the scenario “not insured by AOK at all”, the latter is the case for roughly 2/3 of the german population [https://www.bundesgesundheitsministerium.de/themen/krankenversicherung/zahlen-und-fakten-zur-krankenversicherung/kennzahlen-daten-bekanntmachungen.html].
2. No primary inpatient diagnosis (The primary inpatient diagnosis indicates entity-specific treatment in a hospital; the focus of the analysis was on survival differences between patients with center and non-center treatments. To compare a complex intervention in a meaningful way we require patients to have at least one hospital stay in order to actually compare hospitals with or without certification. In addition, we expect the outpatient cohort to exhibit very different patterns of treatment.)
3. Age < 18 years at diagnosis (Analysis was restricted to the adult population to exclude rare, special cases of colorectal cancer)
4. Washout: Inpatient or outpatient diagnosis of colon or rectal cancer in the period 2006-2008 (Used for identification of incident cases of colorectal cancer)
5. Treatment in a hospital which became a certified center within 1 year before index treatment (Hospitals are likely to have already established structures required for certification before the certificate is issued; patients treated in this 1-year period before certification therefore are likely to have effectively received center treatment)
6. Primary resection more than 6 months after index treatment (Primary tumor resection was used for definition of index treatment only if it occurs within a reasonable time frame after the first relevant inpatient diagnosis of colorectal cancer; other cases may have low plausibility)
7. Survival time of zero (patients dying at the date of index treatment may not benefit from treatment in certified center
8. Missing hospital characteristics (The full regression model adjusts for hospital characteristics that represent potential confounders of center treatment. Patients treated in hospitals for which those characteristics were unknown were excluded to ensure comparability of results between different model specifications, i.e. complete case analysis). The number of patients with the sole exclusion reason being “missing hospital characteristics” for both colon and rectal cancer, split by category (multiple missings per patient are possible) is summarized in the following table (missings are due to incomplete information in data provided by the SQR.):

| missings, category | Colon, n | Rectum, n |
| --- | --- | --- |
| "university hospital" | 1936 | 751 |
| "teaching hospital" | 606 | 238 |
| "hospital type" | 822 | 316 |
| "number of beds" | 606 | 238 |
| total | 2152 | 829 |

### Table S1: OPS-codes for definition of resection (in German):

| OPS:5-455 | Inzision, Exzision, Resektion und Anastomose an Dünn- und Dickdarm- Partielle Resektion des Dickdarmes |
| --- | --- |
| OPS:5-456 | Inzision, Exzision, Resektion und Anastomose an Dünn- und Dickdarm- (Totale) Kolektomie und Proktokolektomie |
| OPS:5-459 | Inzision, Exzision, Resektion und Anastomose an Dünn- und Dickdarm- Bypass-Anastomose des Darmes |
| OPS:5-482 | Operationen am Rektum- Peranale lokale Exzision und Destruktion von erkranktem Gewebe des Rektums |
| OPS:5-484 | Operationen am Rektum- Rektumresektion unter Sphinktererhaltung |
| OPS:5-485 | Operationen am Rektum- Rektumresektion ohne Sphinktererhaltung |
| OPS:5-541.2 | Andere Operationen in der Bauchregion- Laparotomie und Eröffnung des Retroperitoneums- Relaparotomie-NA |
| OPS:5-471 | Operationen an der Appendix- Simultane Appendektomie |

**Figure S1: relative survival stratified by hospital size**

1. **Colon, 1-299 beds B) Colon, 300-499 beds**


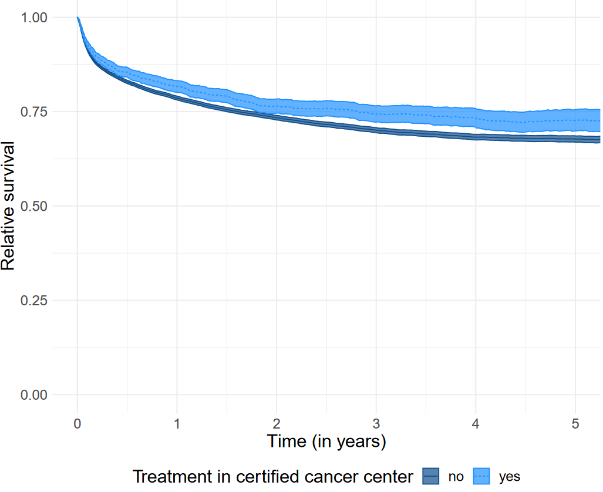

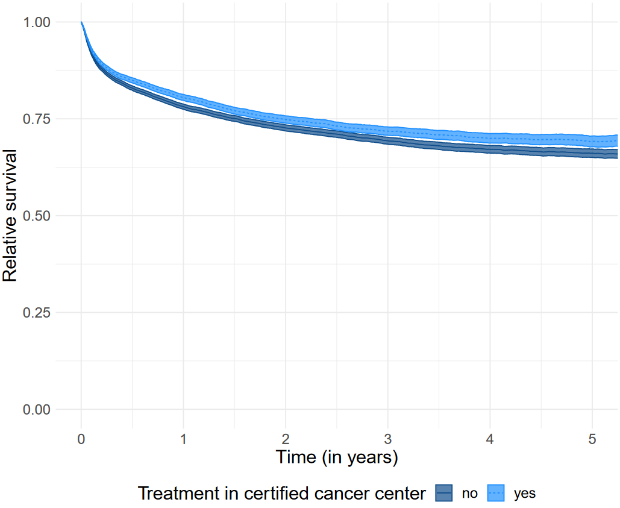


C) Colon, 500-999 beds D) Colon, 1000+ beds


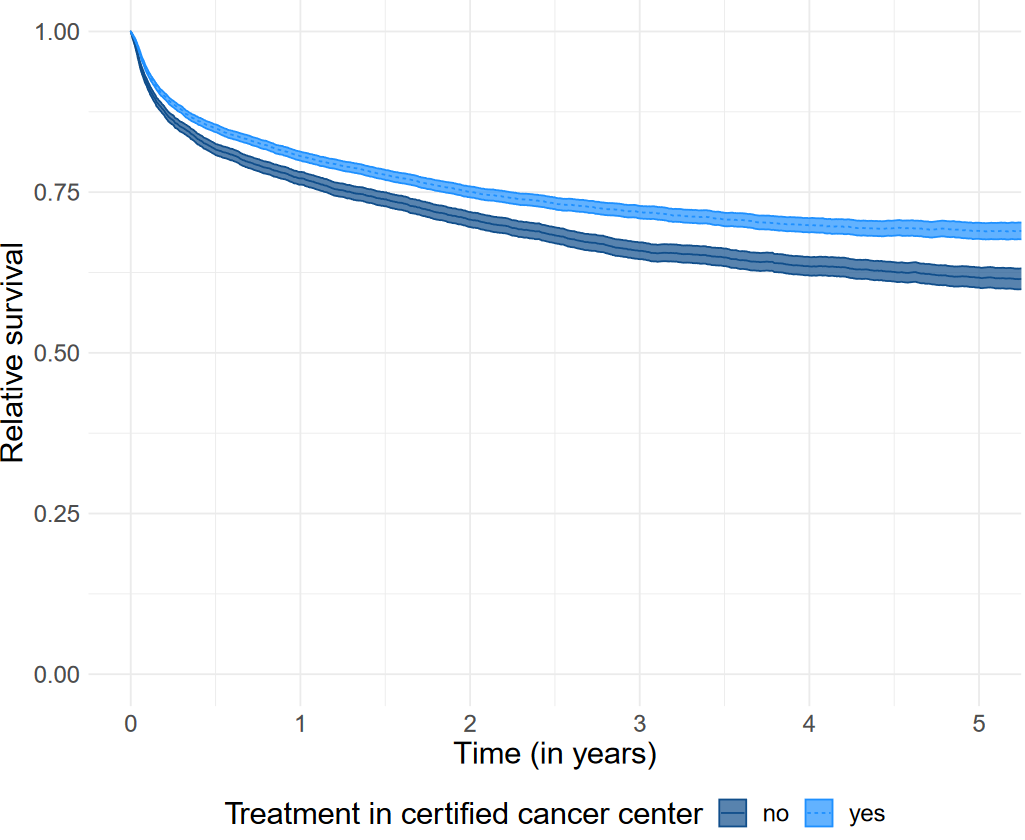

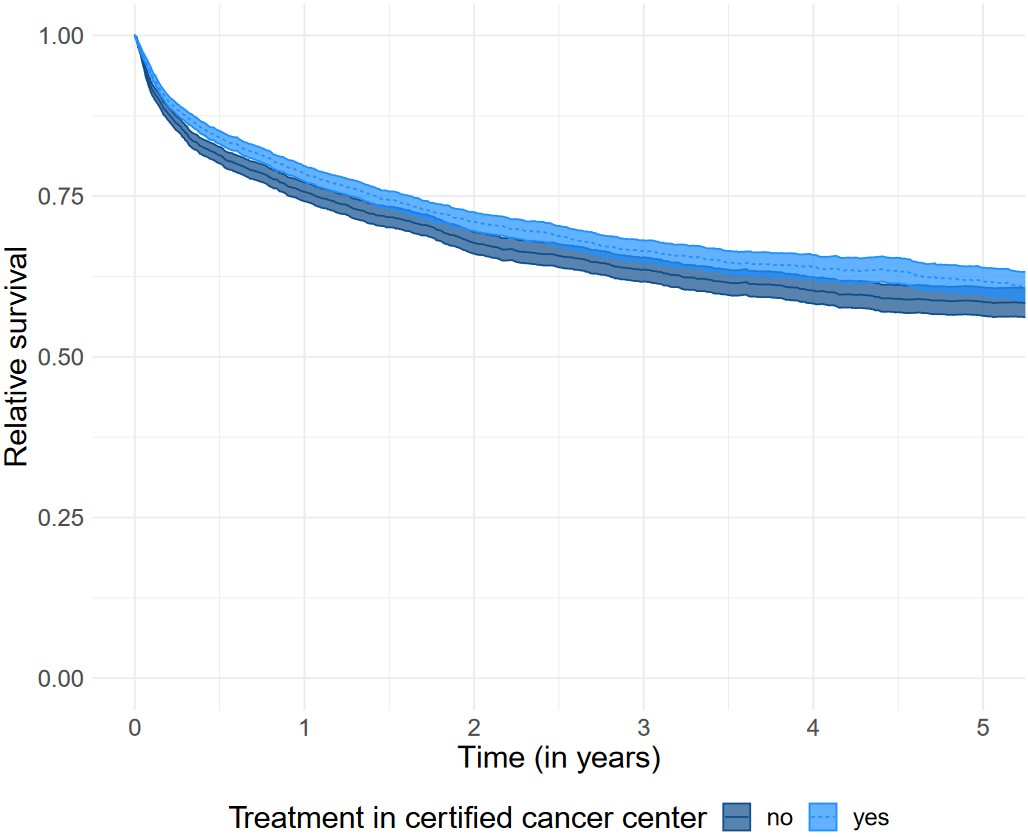


1. **Rectum, 1-299 beds F) Rectum, 300-499 beds**


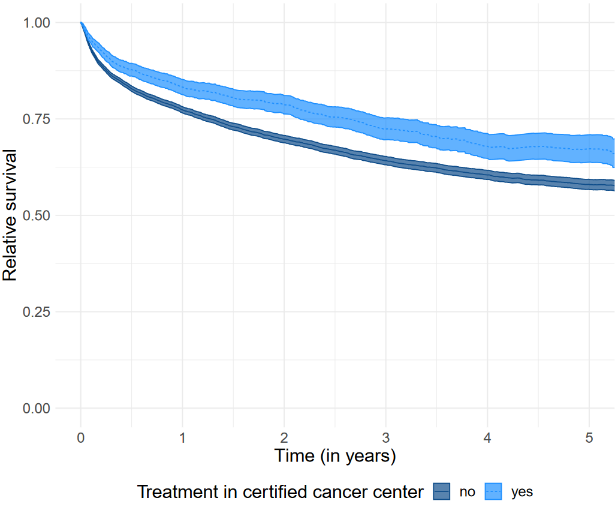

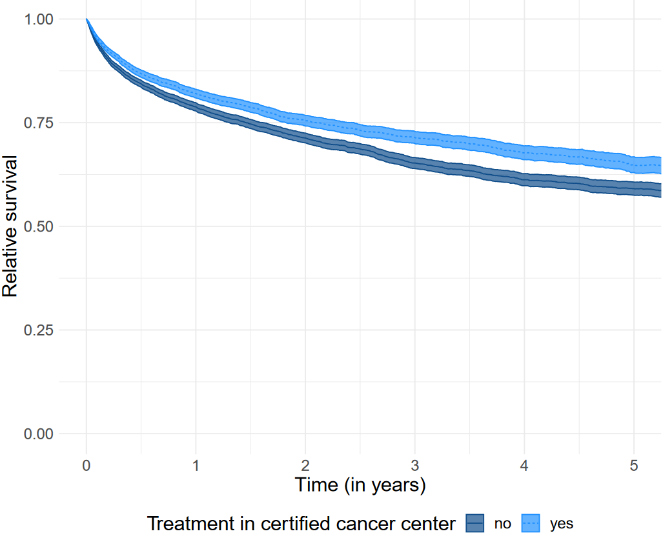


G) Rectum, 500-999 beds H) Rectum, 1000+ beds

**
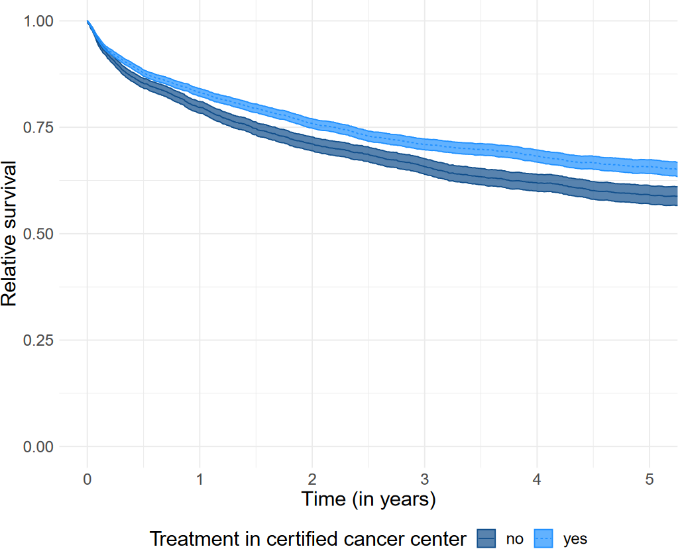

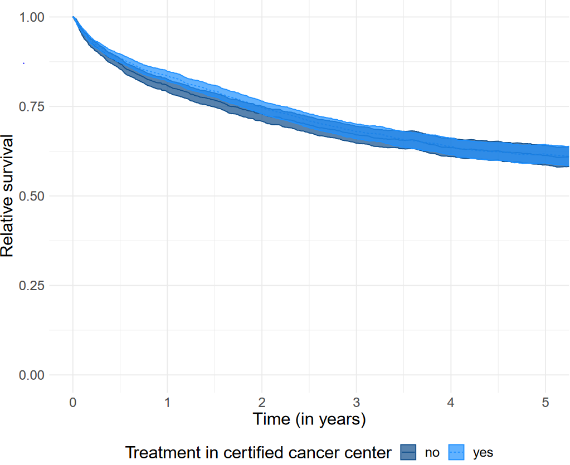
**

**Table S2a: Clopper-Pearson Intervals of baseline table for patients with colon cancer by certification status with interval overlap**

| Variable | cert., % | cert., lower CI, % | cert., upper CI, % | non-cert., % | non-cert., lower CI % | cert., upper CI, % | CI overlap |
| --- | --- | --- | --- | --- | --- | --- | --- |
| Age 18-59 | 12.7% | 12.5 | 13.0 | 11.6% | 11.4 | 11.7 | no |
| Age 60-79 | 56.7% | 56.4 | 57.1 | 0.55 | 54.8 | 55.3 | no |
| Age 80+ | 30.5% | 30.2 | 30.8 | 33.4% | 33.2 | 33.6 | no |
| Sex female | 48.9% | 48.6 | 49.3 | 50.5% | 50.2 | 50.7 | no |
| Sex male | 51.1% | 50.7 | 51.4 | 49.5% | 49.3 | 49.8 | no |
| Distant metastasis yes | 28.8% | 28.5 | 29.1 | 26.4% | 26.2 | 26.7 | no |
| Other oncological disease | 37.4% | 37.1 | 37.7 | 35.8% | 35.6 | 36.1 | no |
| Congestive heart failure | 35.4% | 35.1 | 35.7 | 38.3% | 38.1 | 38.6 | no |
| Cardiac arrhythmias | 38.7% | 38.4 | 39.0 | 38.1% | 37.8 | 38.3 | no |
| Valvular disease | 20.5% | 20.2 | 20.7 | 19.7% | 19.5 | 19.9 | no |
| Pulmonary circulation disorders | 8.6% | 8.4 | 8.7 | 8.1% | 7.9 | 8.2 | no |
| Periph. vascular disorders | 31.2% | 30.9 | 31.5 | 30.5% | 30.3 | 30.7 | no |
| Hypertension (uncomplicated) | 81.7% | 81.4 | 81.9 | 82.7% | 82.5 | 82.9 | no |
| Hypertension (complicated) | 27.6% | 27.3 | 27.9 | 28.2% | 28.0 | 28.5 | no |
| Other neurological disorders | 11.4% | 11.2 | 11.6 | 11.3% | 11.2 | 11.5 | yes |
| Chronic pulmonary disease | 44.8% | 44.5 | 45.1 | 42.5% | 42.3 | 42.8 | no |
| Diabetes (uncomplicated) | 38.8% | 38.5 | 39.1 | 39.2% | 39.0 | 39.5 | yes |
| Diabetes (complicated) | 21.9% | 21.6 | 22.2 | 21.4% | 21.2 | 21.6 | no |
| Renal failure | 25.4% | 25.2 | 25.7 | 24.9% | 24.7 | 25.2 | no |
| Liver disease | 29.7% | 29.4 | 29.9 | 26.8% | 26.5 | 27.0 | no |
| Blood loss anemia | 9.1% | 8.9 | 9.3 | 9.5% | 9.3 | 9.6 | no |
| Deficiency anemia | 27.9% | 27.6 | 28.1 | 26.7% | 26.5 | 27.0 | no |
| Alcohol abuse | 7.2% | 7.0 | 7.4 | 0.07 | 6.9 | 7.1 | yes |
| Drug abuse | 2.3% | 2.2 | 2.4 | 2.5% | 0.2 | 0.3 | no |

**Table S2b: Clopper-Pearson Intervals of baseline table for patients with rectal cancer by certification status with interval overlap**

| Variable | cert., % | cert., lower CI, % | cert., upper CI, % | non-cert., % | non-cert., lower CI % | cert., upper CI, % | CI overlap |
| --- | --- | --- | --- | --- | --- | --- | --- |
| Age 18-59 | 18.8% | 18.5 | 19.1 | 15% | 14.7 | 15.3 | no |
| Age 60-79 | 59.4% | 59.0 | 59.8 | 58.1% | 57.7 | 58.4 | no |
| Age 80+ | 21.8% | 21.5 | 22.2 | 26.9% | 26.6 | 27.2 | no |
| Sex female | 39.1% | 38.7 | 39.5 | 40.8% | 40.5 | 41.2 | no |
| Sex male | 60.9% | 60.5 | 61.3 | 59.2% | 58.8 | 59.5 | no |
| Distant metastasis yes | 25.5% | 25.2 | 25.9 | 25.6% | 25.3 | 25.9 | yes |
| Other oncological disease | 50% | 49.6 | 50.4 | 49.7% | 49.3 | 50.0 | yes |
| Congestive heart failure | 27.6% | 27.3 | 28.0 | 32.6% | 32.2 | 32.9 | no |
| Cardiac arrhythmias | 32.4% | 32.0 | 32.8 | 33.1% | 32.8 | 33.5 | yes |
| Valvular disease | 16.1% | 15.8 | 16.4 | 16.6% | 16.3 | 16.9 | yes |
| Pulmonary circulation disorders | 5.9% | 5.7 | 6.1 | 6.2% | 6.1 | 6.4 | yes |
| Periph. vascular disorders | 30.3% | 29.9 | 30.7 | 30.2% | 29.9 | 30.5 | yes |
| Hypertension (uncomplicated) | 75.8% | 75.4 | 76.2 | 78.5% | 78.2 | 78.8 | no |
| Hypertension (complicated) | 22.7% | 22.3 | 23.1 | 24.7% | 24.4 | 25.0 | no |
| Other neurological disorders | 9.4% | 9.2 | 9.7 | 10.2% | 9.9 | 10.4 | no |
| Chronic pulmonary disease | 40.9% | 40.5 | 41.3 | 38.9% | 38.5 | 39.2 | no |
| Diabetes (uncomplicated) | 34.4% | 34.0 | 34.8 | 36.3% | 35.9 | 36.6 | no |
| Diabetes (complicated) | 18.7% | 18.4 | 19.1 | 19% | 18.7 | 19.3 | yes |
| Renal failure | 19.9% | 19.5 | 20.2 | 20.7% | 20.4 | 21.1 | no |
| Liver disease | 27.8% | 27.4 | 28.2 | 24.9% | 24.6 | 25.2 | no |
| Blood loss anemia | 4.7% | 4.5 | 4.9 | 5.6% | 5.4 | 5.8 | no |
| Deficiency anemia | 15.1% | 14.8 | 15.5 | 15.8% | 15.5 | 16.0 | no |
| Alcohol abuse | 8.9% | 8.6 | 9.1 | 8.8% | 8.5 | 9.0 | yes |
| Drug abuse | 2% | 1.9 | 2.1 | 2.2% | 2.1 | 2.3 | yes |

**Table S3: Baseline table for hospital characteristics for patients with rectal cancer by certification status**

| Variable | All | (n=1,051) | Certified: no | (n=741) | Certified: yes | (n=310) |
| --- | --- | --- | --- | --- | --- | --- |
| Hospital beds, n (%) |  |  |  |  |  |  |
| 1-299 | 580 | (55.2%) | 540 | (72.9%) | 40 | (12.9%) |
| 300-499 | 261 | (24.8%) | 142 | (19.2%) | 119 | (38.4%) |
| 500-999 | 160 | (15.2%) | 50 | (6.7%) | 110 | (35.5%) |
| 1000+ | 50 | (4.8%) | 9 | (1.2%) | 41 | (13.2%) |
| Teaching hospital, n (%) |  |  |  |  |  |  |
| no | 449 | (42.7%) | 399 | (53.8%) | 50 | (16.1%) |
| yes | 602 | (57.3%) | 342 | (46.2%) | 260 | (83.9%) |
| University hospital, n (%) |  |  |  |  |  |  |
| no | 1,023 | (97.3%) | 734 | (99.1%) | 289 | (93.2%) |
| yes | 28 | (2.7%) | 7 | (0.9%) | 21 | (6.8%) |
| Hospital ownership, n (%) |  |  |  |  |  |  |
| public | 382 | (36.3%) | 239 | (32.3%) | 143 | (46.1%) |
| non-profit | 459 | (43.7%) | 336 | (45.3%) | 123 | (39.7%) |
| private | 210 | (20%) | 166 | (22.4%) | 44 | (14.2%) |
| Certified center, n (%) |  |  |  |  |  |  |
| no | 741 | (70.5%) |  |  |  |  |
| yes | 310 | (29.5%) |  |  |  |  |

**Table S4: Overall survival rates for 30days, 1, 2, 3, 4, 5 years with 95% CI-intervals (lower, upper)**

| Entity | certified | 30d | 1year | 2year | 3year | 4year | 5year |
| --- | --- | --- | --- | --- | --- | --- | --- |
| colon | yes | 0.940 (0.937,0.942) | 0.756 (0.751,0.760) | 0.657 (0.652,0.662) | 0.587 (0.582,0.593) | 0.532 (0.527,0.538) | 0.487 (0.481,0.493) |
|  | no | 0.923 (0.921,0.925) | 0.732 (0.729,0.736) | 0.636 (0.632,0.640) | 0.564 (0.560,0.568) | 0.509 (0.505,0.513) | 0.467 (0.463,0.471) |
| Rectum | yes | 0.959 (0.956,0.962) | 0.786 (0.781,0.792) | 0.682 (0.676,0.689) | 0.602 (0.595,0.610) | 0.541 (0.534,0.549) | 0.492 (0.484,0.500) |
|  | no | 0.943 (0.941,0.946) | 0.739 (0.734,0.744) | 0.628 (0.623,0.634) | 0.544 (0.538,0.550) | 0.481 (0.474,0.487) | 0.433 (0.427,0.440) |

### Supplemental Table S5: Full results of Cox regression with shared frailty for colon cancer

| Colon cancer, C18/C19 | | | | | | | | | | |
| --- | --- | --- | --- | --- | --- | --- | --- | --- | --- | --- |
|  | certification only | | + age, sex | | + metastasis, secondary malignoma, Elixhauser comorbidities | | + hospital characteristics | | + calendar year dummies | |
| Variable | HR | CI | HR | CI | HR | CI | HR | CI | HR | CI |
| Certified center (ref: no) | - | - | - | - | - | - | - | - | - | - |
| yes | 0.93*** | (0.90,0.96) | 0.94*** | (0.91,0.96) | 0.89*** | (0.87,0.92) | 0.88*** | (0.86,0.91) | 0.92*** | (0.89,0.95) |
| Age (ref: 18-59) | - | - | - | - | - | - | - | - | - | - |
| 60-79 |  |  | 1.73*** | (1.67,1.79) | 1.62*** | (1.56,1.68) | 1.62*** | (1.56,1.68) | 1.61*** | (1.55,1.67) |
| 80+ |  |  | 3.58*** | (3.45,3.70) | 3.23*** | (3.11,3.36) | 3.23*** | (3.11,3.36) | 3.22*** | (3.10,3.35) |
| Sex (ref: female) | - | - | - | - | - | - | - | - | - | - |
| male |  |  | 1.17*** | (1.15,1.19) | 1.10*** | (1.08,1.12) | 1.10*** | (1.08,1.12) | 1.10*** | (1.08,1.12) |
| Distant metastasis (ref: no) | - | - | - | - | - | - | - | - | - | - |
| yes |  |  |  |  | 4.19*** | (4.11,4.26) | 4.19*** | (4.11,4.26) | 4.20*** | (4.13,4.28) |
| Other oncological disease (ref: no) | - | - | - | - | - | - | - | - | - | - |
| yes |  |  |  |  | 0.98* | (0.96,1.00) | 0.98* | (0.96,1.00) | 0.98* | (0.96,1.00) |
| Congestive heart failure (ref: no) | - | - | - | - | - | - | - | - | - | - |
| yes |  |  |  |  | 1.41*** | (1.38,1.44) | 1.41*** | (1.38,1.44) | 1.40*** | (1.37,1.43) |
| Cardiac arrhythmias (ref: no) | - | - | - | - | - | - | - | - | - | - |
| yes |  |  |  |  | 1.04*** | (1.02,1.06) | 1.04*** | (1.02,1.06) | 1.05*** | (1.03,1.07) |
| Valvular disease (ref: no) | - | - | - | - | - | - | - | - | - | - |
| yes |  |  |  |  | 0.95*** | (0.93,0.97) | 0.95*** | (0.93,0.97) | 0.95*** | (0.93,0.97) |
| Pulmonary circulation disorders (ref: no) | - | - | - | - | - | - | - | - | - | - |
| yes |  |  |  |  | 1.27*** | (1.23,1.31) | 1.27*** | (1.23,1.31) | 1.27*** | (1.23,1.31) |
| Periph. vascular disorders (ref: no) | - | - | - | - | - | - | - | - | - | - |
| yes |  |  |  |  | 1.07*** | (1.05,1.09) | 1.07*** | (1.05,1.09) | 1.08*** | (1.06,1.10) |
| Hypertension, (uc) (ref: no) | - | - | - | - | - | - | - | - | - | - |
| yes |  |  |  |  | 0.92*** | (0.90,0.94) | 0.92*** | (0.90,0.94) | 0.92*** | (0.90,0.95) |
| Hypertension, (c) (ref: no) | - | - | - | - | - | - | - | - | - | - |
| yes |  |  |  |  | 0.91*** | (0.89,0.93) | 0.91*** | (0.89,0.93) | 0.92*** | (0.90,0.94) |
| Other neurological disorders (ref: no) | - | - | - | - | - | - | - | - | - | - |
| yes |  |  |  |  | 1.37*** | (1.33,1.40) | 1.37*** | (1.33,1.40) | 1.37*** | (1.33,1.40) |
| Chronic pulmonary disease (ref: no) | - | - | - | - | - | - | - | - | - | - |
| yes |  |  |  |  | 1.00 | (0.98,1.02) | 1.00 | (0.98,1.02) | 1.01 | (0.99,1.02) |
| Diabetes (uc) (ref: no) | - | - | - | - | - | - | - | - | - | - |
| yes |  |  |  |  | 1.10*** | (1.07,1.12) | 1.10*** | (1.07,1.12) | 1.09*** | (1.07,1.12) |
| Diabetes (c) (ref: no) | - | - | - | - | - | - | - | - | - | - |
| yes |  |  |  |  | 1.03* | (1.00,1.05) | 1.03* | (1.00,1.05) | 1.03** | (1.01,1.06) |
| Renal failure (ref: no) | - | - | - | - | - | - | - | - | - | - |
| yes |  |  |  |  | 1.29*** | (1.26,1.32) | 1.29*** | (1.26,1.32) | 1.30*** | (1.28,1.33) |
| Liver disease (ref: no) | - | - | - | - | - | - | - | - | - | - |
| yes |  |  |  |  | 0.97*** | (0.95,0.99) | 0.97*** | (0.95,0.99) | 0.97** | (0.95,0.99) |
| Blood loss anemia (ref: no) | - | - | - | - | - | - | - | - | - | - |
| yes |  |  |  |  | 1.01 | (0.99,1.04) | 1.01 | (0.99,1.04) | 1.01 | (0.98,1.04) |
| Deficiency anemia (ref: no) | - | - | - | - | - | - | - | - | - | - |
| yes |  |  |  |  | 1.01 | (0.99,1.03) | 1.01 | (0.99,1.03) | 1.02 | (1.00,1.04) |
| Alcohol abuse (ref: no) | - | - | - | - | - | - | - | - | - | - |
| yes |  |  |  |  | 1.39*** | (1.34,1.44) | 1.39*** | (1.34,1.44) | 1.40*** | (1.35,1.44) |
| Drug abuse (ref: no) | - | - | - | - | - | - | - | - | - | - |
| yes |  |  |  |  | 1.16*** | (1.11,1.23) | 1.16*** | (1.11,1.22) | 1.16*** | (1.10,1.22) |
| Hospital beds (ref: 1-299) | - | - | - | - | - | - | - | - | - | - |
| 300-499 |  |  |  |  |  |  | 1.04 | (1.00,1.07) | 1.02 | (0.98,1.05) |
| 500-999 |  |  |  |  |  |  | 1.05* | (1.01,1.10) | 1.03 | (0.98,1.07) |
| 1000+ |  |  |  |  |  |  | 1.08* | (1.01,1.16) | 1.05 | (0.98,1.12) |
| Teaching hospital (ref: no) | - | - | - | - | - | - | - | - | - | - |
| yes |  |  |  |  |  |  | 0.97 | (0.94,1.00) | 0.98 | (0.95,1.01) |
| University hospital (ref: no) | - | - | - | - | - | - | - | - | - | - |
| yes |  |  |  |  |  |  | 0.99 | (0.90,1.08) | 1.00 | (0.91,1.10) |
| Hospital ownership (ref: public) | - | - | - | - | - | - | - | - | - | - |
| non-profit |  |  |  |  |  |  | 1.01 | (0.98,1.04) | 1.01 | (0.98,1.04) |
| private |  |  |  |  |  |  | 1.03 | (0.99,1.07) | 1.03 | (0.99,1.07) |
| Year of index treatment (ref: 2009) | - | - | - | - | - | - | - | - | - | - |
| 2010 |  |  |  |  |  |  |  |  | 0.95** | (0.92,0.98) |
| 2011 |  |  |  |  |  |  |  |  | 0.97 | (0.94,1.01) |
| 2012 |  |  |  |  |  |  |  |  | 0.95** | (0.92,0.98) |
| 2013 |  |  |  |  |  |  |  |  | 0.93*** | (0.90,0.97) |
| 2014 |  |  |  |  |  |  |  |  | 0.90*** | (0.87,0.93) |
| 2015 |  |  |  |  |  |  |  |  | 0.87*** | (0.84,0.90) |
| 2016 |  |  |  |  |  |  |  |  | 0.86*** | (0.82,0.89) |
| 2017 |  |  |  |  |  |  |  |  | 0.82*** | (0.77,0.86) |
| Number of patients | 109,687 |  | 109,687 |  | 109,687 |  | 109,687 |  | 109,687 |  |
| Number of hospitals | 1,087 |  | 1,087 |  | 1,087 |  | 1,087 |  | 1,087 |  |
| SD(RE) | 0.23 |  | 0.2 |  | 0.18 |  | 0.17 |  | 0.18 |  |
| Model | me |  | me |  | me |  | me |  | me |  |

### HR=Hazard ratio, CI=95%-confidence interval, significance levels: *1%, **5%, ***0.1%

### Supplemental Table S6: Full results of Cox regression with shared frailty for rectal cancer

| **Rectal cancer, C20** | | | | | | | | | | |
| --- | --- | --- | --- | --- | --- | --- | --- | --- | --- | --- |
|  | **certification only** | | **+ age, sex** | | **+ metastasis, secondary malignoma, Elixhauser comorbidities** | | **+ hospital characteristics** | | **+ calendar year dummies** | |
| **Variable** | **HR** | **CI** | **HR** | **CI** | **HR** | **CI** | **HR** | **CI** | **HR** | **CI** |
| **Certified center (ref: no)** | **-** | **-** | **-** | **-** | **-** | **-** | **-** | **-** | **-** | **-** |
| **yes** | **0.86***** | **(0.83,0.89)** | **0.88***** | **(0.86,0.91)** | **0.88***** | **(0.85,0.90)** | **0.88***** | **(0.85,0.92)** | **0.90***** | **(0.87,0.94)** |
| **Age (ref: 18-59)** | **-** | **-** | **-** | **-** | **-** | **-** | **-** | **-** | **-** | **-** |
| **60-79** |  |  | **1.76***** | **(1.68,1.83)** | **1.72***** | **(1.65,1.80)** | **1.72***** | **(1.65,1.80)** | **1.71***** | **(1.64,1.79)** |
| **80+** |  |  | **3.79***** | **(3.62,3.97)** | **3.59***** | **(3.42,3.78)** | **3.59***** | **(3.41,3.77)** | **3.58***** | **(3.40,3.76)** |
| **Sex (ref: female)** | **-** | **-** | **-** | **-** | **-** | **-** | **-** | **-** | **-** | **-** |
| **male** |  |  | **1.11***** | **(1.08,1.14)** | **1.05***** | **(1.03,1.08)** | **1.06***** | **(1.03,1.08)** | **1.06***** | **(1.03,1.08)** |
| **Distant metastasis (ref: no)** | **-** | **-** | **-** | **-** | **-** | **-** | **-** | **-** | **-** | **-** |
| **yes** |  |  |  |  | **3.59***** | **(3.50,3.69)** | **3.60***** | **(3.50,3.69)** | **3.61***** | **(3.51,3.70)** |
| **Other oncological disease (ref: no)** | **-** | **-** | **-** | **-** | **-** | **-** | **-** | **-** | **-** | **-** |
| **yes** |  |  |  |  | **0.91***** | **(0.88,0.93)** | **0.91***** | **(0.88,0.93)** | **0.91***** | **(0.88,0.93)** |
| **Congestive heart failure (ref: no)** | **-** | **-** | **-** | **-** | **-** | **-** | **-** | **-** | **-** | **-** |
| **yes** |  |  |  |  | **1.34***** | **(1.30,1.38)** | **1.34***** | **(1.30,1.38)** | **1.33***** | **(1.29,1.38)** |
| **Cardiac arrhythmias (ref: no)** | **-** | **-** | **-** | **-** | **-** | **-** | **-** | **-** | **-** | **-** |
| **yes** |  |  |  |  | **1.03** | **(1.00,1.06)** | **1.03** | **(1.00,1.06)** | **1.04*** | **(1.01,1.07)** |
| **Valvular disease (ref: no)** | **-** | **-** | **-** | **-** | **-** | **-** | **-** | **-** | **-** | **-** |
| **yes** |  |  |  |  | **0.95**** | **(0.92,0.99)** | **0.95**** | **(0.92,0.99)** | **0.95**** | **(0.92,0.99)** |
| **Pulmonary circulation disorders (ref: no)** | **-** | **-** | **-** | **-** | **-** | **-** | **-** | **-** | **-** | **-** |
| **yes** |  |  |  |  | **1.28***** | **(1.22,1.34)** | **1.28***** | **(1.22,1.34)** | **1.28***** | **(1.22,1.35)** |
| **Periph. vascular disorders (ref: no)** | **-** | **-** | **-** | **-** | **-** | **-** | **-** | **-** | **-** | **-** |
| **yes** |  |  |  |  | **1.05***** | **(1.03,1.08)** | **1.05***** | **(1.03,1.08)** | **1.06***** | **(1.03,1.09)** |
| **Hypertension, (uc) (ref: no)** | **-** | **-** | **-** | **-** | **-** | **-** | **-** | **-** | **-** | **-** |
| **yes** |  |  |  |  | **0.91***** | **(0.88,0.95)** | **0.91***** | **(0.88,0.95)** | **0.91***** | **(0.88,0.95)** |
| **Hypertension, (c) (ref: no)** | **-** | **-** | **-** | **-** | **-** | **-** | **-** | **-** | **-** | **-** |
| **yes** |  |  |  |  | **0.91***** | **(0.88,0.94)** | **0.91***** | **(0.88,0.94)** | **0.91***** | **(0.88,0.94)** |
| **Other neurological disorders (ref: no)** | **-** | **-** | **-** | **-** | **-** | **-** | **-** | **-** | **-** | **-** |
| **yes** |  |  |  |  | **1.44***** | **(1.38,1.49)** | **1.44***** | **(1.38,1.49)** | **1.44***** | **(1.39,1.50)** |
| **Chronic pulmonary disease (ref: no)** | **-** | **-** | **-** | **-** | **-** | **-** | **-** | **-** | **-** | **-** |
| **yes** |  |  |  |  | **0.97*** | **(0.95,1.00)** | **0.97** | **(0.95,1.00)** | **0.98** | **(0.95,1.01)** |
| **Diabetes (uc) (ref: no)** | **-** | **-** | **-** | **-** | **-** | **-** | **-** | **-** | **-** | **-** |
| **yes** |  |  |  |  | **1.09***** | **(1.06,1.13)** | **1.09***** | **(1.06,1.13)** | **1.09***** | **(1.05,1.12)** |
| **Diabetes (c) (ref: no)** | **-** | **-** | **-** | **-** | **-** | **-** | **-** | **-** | **-** | **-** |
| **yes** |  |  |  |  | **1.02** | **(0.98,1.06)** | **1.02** | **(0.98,1.06)** | **1.03** | **(0.99,1.07)** |
| **Renal failure (ref: no)** | **-** | **-** | **-** | **-** | **-** | **-** | **-** | **-** | **-** | **-** |
| **yes** |  |  |  |  | **1.26***** | **(1.22,1.30)** | **1.26***** | **(1.22,1.30)** | **1.27***** | **(1.23,1.31)** |
| **Liver disease (ref: no)** | **-** | **-** | **-** | **-** | **-** | **-** | **-** | **-** | **-** | **-** |
| **yes** |  |  |  |  | **0.95***** | **(0.92,0.98)** | **0.95***** | **(0.92,0.98)** | **0.95**** | **(0.93,0.98)** |
| **Blood loss anemia (ref: no)** | **-** | **-** | **-** | **-** | **-** | **-** | **-** | **-** | **-** | **-** |
| **yes** |  |  |  |  | **1.19***** | **(1.13,1.25)** | **1.19***** | **(1.13,1.25)** | **1.19***** | **(1.13,1.25)** |
| **Deficiency anemia (ref: no)** | **-** | **-** | **-** | **-** | **-** | **-** | **-** | **-** | **-** | **-** |
| **yes** |  |  |  |  | **1.19***** | **(1.15,1.23)** | **1.19***** | **(1.15,1.23)** | **1.20***** | **(1.16,1.24)** |
| **Alcohol abuse (ref: no)** | **-** | **-** | **-** | **-** | **-** | **-** | **-** | **-** | **-** | **-** |
| **yes** |  |  |  |  | **1.52***** | **(1.45,1.58)** | **1.52***** | **(1.45,1.58)** | **1.53***** | **(1.46,1.60)** |
| **Drug abuse (ref: no)** | **-** | **-** | **-** | **-** | **-** | **-** | **-** | **-** | **-** | **-** |
| **yes** |  |  |  |  | **1.21***** | **(1.12,1.31)** | **1.21***** | **(1.12,1.31)** | **1.21***** | **(1.12,1.30)** |
| **Hospital beds (ref: 1-299)** | **-** | **-** | **-** | **-** | **-** | **-** | **-** | **-** | **-** | **-** |
| **300-499** |  |  |  |  |  |  | **1.00** | **(0.96,1.04)** | **0.98** | **(0.94,1.03)** |
| **500-999** |  |  |  |  |  |  | **0.99** | **(0.94,1.04)** | **0.97** | **(0.92,1.02)** |
| **1000+** |  |  |  |  |  |  | **1.00** | **(0.93,1.07)** | **0.98** | **(0.91,1.05)** |
| **Teaching hospital (ref: no)** | **-** | **-** | **-** | **-** | **-** | **-** | **-** | **-** | **-** | **-** |
| **yes** |  |  |  |  |  |  | **0.99** | **(0.95,1.03)** | **1.00** | **(0.96,1.04)** |
| **University hospital (ref: no)** | **-** | **-** | **-** | **-** | **-** | **-** | **-** | **-** | **-** | **-** |
| **yes** |  |  |  |  |  |  | **0.95** | **(0.86,1.04)** | **0.95** | **(0.86,1.04)** |
| **Hospital ownership (ref: public)** | **-** | **-** | **-** | **-** | **-** | **-** | **-** | **-** | **-** | **-** |
| **non-profit** |  |  |  |  |  |  | **1.01** | **(0.97,1.05)** | **1.01** | **(0.97,1.04)** |
| **private** |  |  |  |  |  |  | **1.02** | **(0.97,1.07)** | **1.02** | **(0.98,1.07)** |
| **Year of index treatment (ref: 2009)** | **-** | **-** | **-** | **-** | **-** | **-** | **-** | **-** | **-** | **-** |
| **2010** |  |  |  |  |  |  |  |  | **1.02** | **(0.97,1.06)** |
| **2011** |  |  |  |  |  |  |  |  | **1.00** | **(0.95,1.04)** |
| **2012** |  |  |  |  |  |  |  |  | **0.97** | **(0.92,1.01)** |
| **2013** |  |  |  |  |  |  |  |  | **0.99** | **(0.94,1.04)** |
| **2014** |  |  |  |  |  |  |  |  | **0.94*** | **(0.89,0.99)** |
| **2015** |  |  |  |  |  |  |  |  | **0.87***** | **(0.82,0.92)** |
| **2016** |  |  |  |  |  |  |  |  | **0.88***** | **(0.83,0.94)** |
| **2017** |  |  |  |  |  |  |  |  | **0.83***** | **(0.76,0.89)** |
| **Number of patients** | **51,456** |  | **51,456** |  | **51,456** |  | **51,456** |  | **51,456** |  |
| **Number of hospitals** | **1,050** |  | **1,050** |  | **1,050** |  | **1,050** |  | **1,050** |  |
| **SD(RE)** | **0.24** |  | **0.14** |  | **0.14** |  | **0.14** |  | **0.14** |  |
| **Model** | **me** |  | **me** |  | **me** |  | **me** |  | **me** |  |

HR=Hazard ratio, CI=95%-confidence interval, significance levels: *1%, **5%, ***0.1%

**Table S7: Hazard ratios (HR) with 95%-confidence intervals (CI) from Cox regressions with shared frailty for certification effect and each hospital characteristic separately (1a-d), and a full model with standard Cox regression (1f).**

|  |  | Colon |  | Rectum |  |
| --- | --- | --- | --- | --- | --- |
| Model nr. | Variable | HR | CI | HR | CI |
| 1a | Certified center yes (ref: no) | 0.92*** | (0.89,0.95) | 0.92*** | (0.89,0.95) |
|  | Hospital beds (ref: 1-299) | - | - | - | - |
|  | 300-499 | 1.00 | (0.96,1.04) | 1.00 | (0.96,1.04) |
|  | 500-999 | 1.01 | (0.97,1.06) | 1.01 | (0.97,1.06) |
|  | 1000+ | 1.07 | (1.00,1.14) | 1.07 | (1.00,1.14) |
| 1b | Certified center yes (ref: no) | 0.92*** | (0.89,0.95) | 0.92*** | (0.89,0.95) |
|  | Teaching hospital yes (ref:no) | 1.01 | (0.98,1.05) | 1.01 | (0.98,1.05) |
| 1c | Certified center yes (ref: no) | 0.92*** | (0.90,0.95) | 0.92*** | (0.90,0.95) |
|  | University hospital yes (ref:no) | 1.03 | (0.93,1.14) | 1.03 | (0.93,1.14) |
| 1d | Certified center yes (ref: no) | 0.93*** | (0.90,0.95) | 0.93*** | (0.90,0.95) |
|  | Type (ref:public): non-profit | 0.98 | (0.95,1.02) | 0.98 | (0.95,1.02) |
|  | Type: private | 1.03 | (0.98,1.08) | 1.03 | (0.98,1.08) |

**Tables S8: Colon cancer:** **Hazard ratios (HR) with 95%-confidence intervals (CI) from Cox regressions with shared frailty for different subgroups of patients and hospitals**

| Colon, C18/C19 | Sex | | | | Secondary malignoma | | | | Distant metastasis | | | | Hospital number | | | |
| --- | --- | --- | --- | --- | --- | --- | --- | --- | --- | --- | --- | --- | --- | --- | --- | --- |
|  | male | | female | | no | | yes | | no | | yes | | single | | association | |
| Variable | HR | CI | HR | CI | HR | CI | HR | CI | HR | CI | HR | CI | HR | CI | HR | CI |
| Certified center (ref: no) | - | - | - | - | - | - | - | - | - | - | - | - | - | - | - | - |
| yes | 0.90*** | (0.87,0.94) | 0.92*** | (0.88,0.95) | 0.92*** | (0.88,0.95) | 0.91*** | (0.86,0.95) | 0.90*** | (0.86,0.93) | 0.93*** | (0.89,0.96) | 0.91*** | (0.88,0.95) | 0.92* | (0.86,0.99) |
| Age (ref: 18-59) | - | - | - | - | - | - | - | - | - | - | - | - | - | - | - | - |
| 60-79 | 1.59*** | (1.52,1.66) | 1.66*** | (1.56,1.75) | 1.67*** | (1.61,1.74) | 1.32*** | (1.21,1.44) | 2.33*** | (2.19,2.48) | 1.30*** | (1.24,1.36) | 1.62*** | (1.55,1.68) | 1.59*** | (1.46,1.73) |
| 80+ | 3.05*** | (2.89,3.21) | 3.46*** | (3.26,3.67) | 3.44*** | (3.29,3.59) | 2.44*** | (2.23,2.67) | 5.30*** | (4.98,5.65) | 2.17*** | (2.07,2.29) | 3.24*** | (3.10,3.38) | 3.16*** | (2.90,3.45) |
| Sex (ref: female) | - | - | - | - | - | - | - | - | - | - | - | - | - | - | - | - |
| male | - | - | - | - | 1.10*** | (1.07,1.12) | 1.10*** | (1.06,1.14) | 1.16*** | (1.13,1.18) | 1.02 | (0.99,1.05) | 1.10*** | (1.08,1.13) | 1.07** | (1.02,1.11) |
| Distant metastasis (ref: no) | - | - | - | - | - | - | - | - | - | - | - | - | - | - | - | - |
| yes | 4.17*** | (4.07,4.28) | 4.27*** | (4.16,4.38) | 4.52*** | (4.43,4.62) | 3.35*** | (3.23,3.48) | - | - | - | - | 4.21*** | (4.13,4.30) | 4.18*** | (4.02,4.35) |
| Other oncological disease (ref: no) | - | - | - | - | - | - | - | - | - | - | - | - | - | - | - | - |
| yes | 0.98 | (0.95,1.01) | 0.98 | (0.95,1.01) | - | - | - | - | 1.08*** | (1.05,1.11) | 0.89*** | (0.86,0.92) | 0.97* | (0.95,0.99) | 0.99 | (0.95,1.04) |
| Year of index treatment (ref: 2009) | - | - | - | - | - | - | - | - | - | - | - | - | - | - | - | - |
| 2010 | 0.95* | (0.91,0.99) | 0.96 | (0.92,1.01) | 0.96* | (0.92,0.99) | 0.94 | (0.88,1.00) | 0.97 | (0.93,1.00) | 0.92*** | (0.87,0.96) | 0.96** | (0.93,0.99) | 0.92* | (0.85,1.00) |
| 2011 | 0.96 | (0.92,1.01) | 0.99 | (0.95,1.04) | 0.99 | (0.95,1.03) | 0.94 | (0.88,1.00) | 0.98 | (0.94,1.02) | 0.94* | (0.89,0.99) | 0.97 | (0.94,1.00) | 0.97 | (0.89,1.05) |
| 2012 | 0.97 | (0.92,1.01) | 0.94* | (0.90,0.99) | 0.96* | (0.92,0.99) | 0.94 | (0.88,1.01) | 0.92*** | (0.88,0.96) | 0.95 | (0.90,1.00) | 0.95** | (0.92,0.99) | 0.90* | (0.83,0.98) |
| 2013 | 0.94** | (0.89,0.98) | 0.94* | (0.90,0.99) | 0.94** | (0.91,0.98) | 0.92* | (0.86,0.99) | 0.92*** | (0.88,0.96) | 0.92** | (0.87,0.97) | 0.95** | (0.91,0.99) | 0.85*** | (0.78,0.93) |
| 2014 | 0.87*** | (0.83,0.92) | 0.94* | (0.89,0.98) | 0.90*** | (0.86,0.94) | 0.89** | (0.83,0.96) | 0.88*** | (0.83,0.92) | 0.89*** | (0.85,0.94) | 0.92*** | (0.88,0.95) | 0.81*** | (0.75,0.88) |
| 2015 | 0.87*** | (0.82,0.92) | 0.88*** | (0.84,0.93) | 0.88*** | (0.84,0.92) | 0.86*** | (0.79,0.93) | 0.87*** | (0.82,0.91) | 0.86*** | (0.81,0.90) | 0.88*** | (0.84,0.92) | 0.81*** | (0.74,0.88) |
| 2016 | 0.84*** | (0.79,0.89) | 0.89*** | (0.84,0.94) | 0.86*** | (0.82,0.91) | 0.85*** | (0.78,0.93) | 0.80*** | (0.76,0.85) | 0.88*** | (0.83,0.93) | 0.86*** | (0.82,0.90) | 0.80*** | (0.73,0.88) |
| 2017 | 0.84*** | (0.78,0.90) | 0.80*** | (0.75,0.87) | 0.82*** | (0.78,0.88) | 0.80*** | (0.72,0.89) | 0.76*** | (0.70,0.82) | 0.86*** | (0.80,0.93) | 0.83*** | (0.78,0.88) | 0.75*** | (0.67,0.84) |
| Number of patients | 54,934 |  | 54,753 |  | 86,474 |  | 23,213 |  | 79,730 |  | 29,957 |  | 85,007 |  | 24,680 |  |
| Number of hospitals | 1,051 |  | 1,062 |  | 1,070 |  | 1,036 |  | 1,059 |  | 1,039 |  | 1,020 |  | 220 |  |

Note: The full set of covariates is included in the model but not shown in the table; HR=Hazard ratio, CI=95%-confidence interval, significance levels: *1%, **5%, ***0.1%

**Table S9: Rectal cancer:** **Hazard ratios (HR) with 95%-confidence intervals (CI) from Cox regressions with shared frailty for different subgroups of patients and hospitals**

| Rectum, C20 | Sex | | | | Secondary malignoma | | | | Distant metastasis | | | | Hospital number | | | |
| --- | --- | --- | --- | --- | --- | --- | --- | --- | --- | --- | --- | --- | --- | --- | --- | --- |
|  | male | | female | | no | | yes | | no | | yes | | single | | association | |
| Variable | HR | CI | HR | CI | HR | CI | HR | CI | HR | CI | HR | CI | HR | CI | HR | CI |
| Certified center (ref: no) | - | - | - | - | - | - | - | - | - | - | - | - | - | - | - | - |
| yes | 0.90*** | (0.86,0.94) | 0.91*** | (0.87,0.96) | 0.89*** | (0.85,0.93) | 0.94* | (0.88,0.99) | 0.91*** | (0.87,0.96) | 0.89*** | (0.84,0.94) | 0.91*** | (0.87,0.94) | 0.88** | (0.81,0.96) |
| Age (ref: 18-59) | - | - | - | - | - | - | - | - | - | - | - | - | - | - | - | - |
| 60-79 | 1.69*** | (1.60,1.78) | 1.77*** | (1.63,1.91) | 1.74*** | (1.65,1.83) | 1.63*** | (1.48,1.79) | 2.10*** | (1.97,2.25) | 1.44*** | (1.35,1.53) | 1.72*** | (1.63,1.80) | 1.69*** | (1.53,1.87) |
| 80+ | 3.43*** | (3.22,3.66) | 3.81*** | (3.51,4.15) | 3.68*** | (3.47,3.89) | 3.35*** | (3.02,3.71) | 4.96*** | (4.62,5.32) | 2.41*** | (2.23,2.59) | 3.59*** | (3.40,3.80) | 3.54*** | (3.17,3.96) |
| Sex (ref: female) | - | - | - | - | - | - | - | - | - | - | - | - | - | - | - | - |
| male | - | - | - | - | 1.06*** | (1.02,1.09) | 1.06* | (1.00,1.11) | 1.08*** | (1.05,1.12) | 1.01 | (0.96,1.05) | 1.07*** | (1.03,1.10) | 1.02 | (0.96,1.08) |
| Distant metastasis (ref: no) | - | - | - | - | - | - | - | - | - | - | - | - | - | - | - | - |
| yes | 3.68*** | (3.56,3.81) | 3.52*** | (3.37,3.67) | 3.79*** | (3.67,3.91) | 3.21*** | (3.06,3.38) | - | - | - | - | 3.61*** | (3.50,3.72) | 3.61*** | (3.40,3.82) |
| Other oncological disease (ref: no) | - | - | - | - | - | - | - | - | - | - | - | - | - | - | - | - |
| yes | 0.91*** | (0.87,0.94) | 0.91*** | (0.87,0.95) | - | - | - | - | 0.95** | (0.91,0.98) | 0.89*** | (0.85,0.93) | 0.91*** | (0.88,0.94) | 0.90** | (0.85,0.96) |
| Year of index treatment (ref: 2009) | - | - | - | - | - | - | - | - | - | - | - | - | - | - | - | - |
| 2010 | 0.99 | (0.93,1.05) | 1.06 | (0.99,1.14) | 1.02 | (0.96,1.07) | 01. Jan | (0.93,1.11) | 1.00 | (0.95,1.06) | 1.03 | (0.95,1.11) | 1.01 | (0.96,1.06) | 1.06 | (0.93,1.20) |
| 2011 | 0.98 | (0.92,1.04) | 1.02 | (0.95,1.10) | 0.99 | (0.94,1.04) | 01. Feb | (0.93,1.11) | 0.99 | (0.94,1.05) | 1.00 | (0.92,1.08) | 1.00 | (0.95,1.05) | 0.99 | (0.88,1.12) |
| 2012 | 0.97 | (0.91,1.03) | 0.97 | (0.90,1.04) | 0.95 | (0.90,1.01) | 0.99 | (0.91,1.09) | 0.97 | (0.91,1.03) | 0.95 | (0.87,1.02) | 0.97 | (0.92,1.02) | 0.97 | (0.86,1.10) |
| 2013 | 0.98 | (0.91,1.04) | 1.01 | (0.94,1.09) | 1.01 | (0.96,1.07) | 0.94 | (0.85,1.03) | 0.99 | (0.93,1.05) | 0.97 | (0.90,1.06) | 0.98 | (0.93,1.03) | 1.04 | (0.92,1.18) |
| 2014 | 0.94 | (0.88,1.00) | 0.94 | (0.87,1.02) | 0.94 | (0.89,1.00) | 0.92 | (0.83,1.01) | 0.91** | (0.85,0.98) | 0.96 | (0.88,1.04) | 0.94* | (0.88,0.99) | 0.95 | (0.84,1.08) |
| 2015 | 0.88*** | (0.82,0.94) | 0.87** | (0.80,0.95) | 0.89*** | (0.83,0.95) | 0.83*** | (0.75,0.93) | 0.87*** | (0.80,0.93) | 0.89** | (0.82,0.97) | 0.88*** | (0.82,0.93) | 0.88 | (0.77,1.01) |
| 2016 | 0.84*** | (0.77,0.91) | 0.94 | (0.86,1.03) | 0.88*** | (0.82,0.95) | 0.86* | (0.77,0.97) | 0.89** | (0.82,0.97) | 0.87** | (0.79,0.96) | 0.86*** | (0.80,0.92) | 0.95 | (0.82,1.09) |
| 2017 | 0.83*** | (0.75,0.92) | 0.83** | (0.73,0.94) | 0.82*** | (0.75,0.90) | 0.82** | (0.71,0.95) | 0.81*** | (0.73,0.90) | 0.85** | (0.76,0.95) | 0.80*** | (0.73,0.88) | 0.90 | (0.76,1.06) |
| Number of patients | 30,835 |  | 20,621 |  | 37,949 |  | 13,507 |  | 38,294 |  | 13,162 |  | 40,144 |  | 11,312 |  |
| Number of hospitals | 1,019 |  | 1,015 |  | 1,038 |  | 975 |  | 1,022 |  | 991 |  | 984 |  | 219 |  |

Note: The full set of covariates is included in the model but not shown in the table; HR=Hazard ratio, CI=95%-confidence interval, significance levels: *5%, **1%, ***0.1%

**Table S10: Descriptive statistics of subgroups of patients with resection date/date of first diagnosis within a given time span from date of index treatment**

| **Colon** | all, n | all, % | cert., n | cert., % | non-cert. , n | non-cert., % |
| --- | --- | --- | --- | --- | --- | --- |
| **Index date vs. resection date** |  |  |  |  |  |  |
| all patients | 109687 | 100.0 | 40861 | 100.0 | 68826 | 100.0 |
| patients with surgery | 89726 | 81.8 | 34422 | 84.2 | 55304 | 80.4 |
| surgery within 4 weeks of index treatment | 86983 | 79.3 | 33017 | 80.8 | 53966 | 78.4 |
| surgery date = date of index treatment | 79536 | 72.5 | 29677 | 72.6 | 49859 | 72.4 |
| **Index date vs. date of diagnosis** |  |  |  |  |  |  |
| diagnosed within 4 weeks of ind. treatment | 98137 | 89.5 | 35868 | 87.8 | 62269 | 90.5 |
| diagnosis date = date of index treatment | 73200 | 66.7 | 25028 | 61.3 | 48172 | 70.0 |
|  |  |  |  |  |  |  |
| **Rectum** |  |  |  |  |  |  |
| **Index date vs. resection date** |  |  |  |  |  |  |
| all patients | 51456 | 100.0 | 22086 | 100.0 | 29370 | 100.0 |
| patients with surgery | 38678 | 75.2 | 17408 | 78.8 | 21270 | 72.4 |
| surgery within 4 weeks of index treatment | 26357 | 51.2 | 11054 | 50.0 | 15303 | 52.1 |
| surgery date = date of index treatment | 22604 | 43.9 | 9257 | 41.9 | 13347 | 45.4 |
| **Index date vs. date of diagnosis** |  |  |  |  |  |  |
| diagnosed within 4 weeks of ind. treatment | 43306 | 84.2 | 17876 | 80.9 | 25430 | 86.6 |
| diagnosis date = date of index treatment | 29880 | 58.1 | 11425 | 51.7 | 18455 | 62.8 |

**Table S11: Hazard ratios (HR) with 95%-confidence intervals (CI) from Cox regressions with shared frailty for resection/no resection and analysis with “date of first diagnosis” instead of “date of index treatment”**

|  | Colon cancer, C18/C19 | | | Rectal cancer, C20 | | |
| --- | --- | --- | --- | --- | --- | --- |
| Variable | number of patients | HR | CI | number of patients | HR | CI |
| Resection only | 89726 | 0.92*** | (0.89,0.95) | 38678 | 0.90*** | (0.86,0.94) |
| no resection | 19961 | 0.96 | (0.90,1.03) | 12778 | 1.04 | (0.99,1.10) |
|  |  |  |  |  |  |  |
| date of first diagnosis | 109687 | 0.91*** | (0.88,0.94) | 51456 | 0.90*** | (0.87,0.94) |

Note: The full set of covariates is included in the model but not shown in the table; HR=Hazard ratio, CI=95%-confidence interval, significance levels: *5%, **1%, ***0.1%

**Table S12: Hazard ratios (HR) with 95%-confidence intervals (CI) from Cox regressions with shared frailty including continuity of certification**

|  | Colon cancer, C18/C19 | | Rectal cancer, C20 | |
| --- | --- | --- | --- | --- |
| Variable | HR | CI | HR | CI |
| Continuity of certification (ref: not certified) | - | - | - | - |
| <1 year | 0.96 | (0.92,1.01) | 0.93* | (0.88,0.99) |
| 1-<2 years | 0.91*** | (0.88,0.96) | 0.93** | (0.88,0.98) |
| 2-<5 years | 0.89*** | (0.86,0.93) | 0.89*** | (0.86,0.93) |
| 5 or more years | 0.90*** | (0.86,0.94) | 0.86*** | (0.82,0.91) |

Note: The full set of covariates is included in the model but not shown in the table; HR=Hazard ratio, CI=95%-confidence interval, significance levels: *5%, **1%, ***0.1%
